# The Risk of COVID-19 Related Hospitalsation, Intensive Care Unit Admission and Mortality in People With Underlying Asthma or COPD: A Systematic Review and Meta-Analysis

**DOI:** 10.3389/fmed.2021.668808

**Published:** 2021-06-16

**Authors:** Shahina Pardhan, Samantha Wood, Megan Vaughan, Mike Trott

**Affiliations:** ^1^Faculty of Health, Education, Medicine and Social Care, School of Medicine, Vision and Eye Research Institute (VERI), Anglia Ruskin University, Cambridge, United Kingdom; ^2^Anglia Ruskin University, Cambridge, United Kingdom

**Keywords:** COVID-19, COPD, asthma, mortality, hospitalsation, meta-analysis, ICU, intensive care

## Abstract

**Background:** Several underlying diseases have been associated with unfavorable COVID-19 related outcomes including asthma and Chronic Obstructive Pulmonary Disease (COPD), however few studies have reported risks that are adjusted for confounding variables. This study aimed to examine the adjusted risk of COVID-19 related hospitalsation, intensive care unit (ICU) admission, and mortality in patients with vs. without asthma or COPD.

**Methods:** A systematic review of major databases was undertaken for studies published between 1/12/2019 and 19/4/2021. Studies reporting the adjusted (for one or more confounder) risks of either hospitalsation, ICU admission, or mortality in asthmatics or COPD patients (control group = no asthma or no COPD) were identified. Risk of bias was determined via the QUIPS tool. A random effect meta-analysis was undertaken.

**Findings:** 37 studies were eligible for analysis, with a total of 1,678,992 participants. The pooled ORs of COVID-19 hospitalsation in subjects with asthma and COPD was 0.91 (95% CI 0.76–1.09) and 1.37 (95% CI 1.29–1.46), respectively. For ICU admission, OR in subjects with asthma and COPD was 0.89 (95% CI 0.74–1.07) and 1.22 (95% CI 1.04–1.42), respectively. For mortality, ORs were 0.88 (95% CI 0.77–1.01) and 1.25 (95% CI 1.08–1.34) for asthma and COPD, respectively. Further, the pooled risk of mortality as measured via Cox regression was 0.93 (95% CI 0.87–1.00) for asthma and 1.30 (95% CI 1.17–1.44) for COPD. All of these findings were of a moderate level of certainty.

**Interpretation:** COPD was significantly associated with COVID-19 related hospital admission, ICU admission, and mortality. Asthma was not associated with negative COVID-19 related health outcomes. Individuals with COPD should take precautions to limit the risk of COVID-19 exposure to negate these potential outcomes. Limitations include differing population types and adjustment for differing cofounding variables. Practitioners should note these findings when dealing with patients with these comorbidities.

**Review Protocol Registration:** https://www.crd.york.ac.uk/prospero/.

## Introduction

In March 2020, the World Health Organization (WHO) declared the COVID-19 outbreak a global pandemic, and as of 3rd February 2021, over 103,000,000 confirmed cases have been diagnosed in more than 130 countries and areas, resulting in ~2,238,000 deaths to date (1). Several risk factors associated with increasing severity of the disease have been reported, including age (2), obesity (3), and underlying conditions such as hypertension (4), and diabetes (5).

An important risk factor for unfavorable COVID-19 outcomes is Chronic Obstructive Pulmonary Disease (COPD); a group of lung conditions including emphysema and chronic bronchitis (6), primarily caused by tobacco smoking, with air pollution, genetic factors, diet and tuberculosis also contributing to the disease (7).

COPD has been associated with increased risks of unfavorable outcomes in non-COVID-19 related pneumonia (8). For COVID-19, some primary studies have questioned whether COPD is associated with worse outcomes (9), whilst the majority of reviews conclude that COPD patients yield significantly worse outcomes than those without (10–13) and others report no effects (14).

An additional risk factor for COVID-19 related complications is the presence of asthma, a common allergy that can cause breathing difficulties including coughing, wheezing, breathlessness and a tight chest (15). Asthma exacerbations have been shown to be strongly associated with other respiratory viral infections, including previous coronaviruses (16, 17). Although some primary studies have reported associations between asthma and negative COVID-19 outcomes, the majority of reviews that have examined associations of COVID-19 outcomes and asthma have concluded a lack of association between asthma and negative COVID-19 outcomes (18, 19).

One limitation of all of the systematic reviews, to date, on COVID-19 outcomes and asthma or COPD is that they report on risk that has not been adjusted for any potential confounding factors, making the true risks of these comorbidities, and subsequent clinical implications, difficult (20)—indeed, of the 16 similar meta-analyses that were published in 2021 (as of April 2021), none of them reported exclusively on adjusted risks; they either report unadjusted risks or the inclusion of adjusted or unadjusted risks is unclear. Several primary studies report on adjusted risks that are lower than the unadjusted risks in several COVID-19 related outcomes, including in asthma (21) and COPD (22). Furthermore, several studies advocate the use of pooling adjusted effect sizes (23, 24), especially in the case of determining COVID-19 related risks (20, 25).

The aim of this review was to examine the risks of negative COVID-19 outcomes in subjects with asthma or COPD, that have been adjusted for one or more COVID-19 related risk factor, including age, sex, smoking status (20, 25), or comorbid disease. Specifically our aims were to assess:

1. Adjusted risk of COVID-19 related hospitalsation in subjects with vs. without asthma or COPD.
2. Adjusted risk of COVID-19 related intensive care unit (ICU) admission in subjects with vs. without asthma or COPD.
3. Adjusted risk of COVID-19 related overall mortality in subjects with vs. without asthma or COPD.

This review has the potential to inform clinicians regarding the true risks of unfavorable COVID-19 outcomes in patients with asthma and COPD, increase awareness in people of the potential risks should they contract COVID-19 and to inform healthcare and public health policies.

## Methods

### Study Registration

This systematic review was conducted in accordance with the Preferred Reporting Items for Systematic Reviews and Meta-Analyses (PRISMA) guidelines (26), and was registered on 29th June 2020 with the international prospective register of systematic reviews (PROSPERO: protocol ID CRD42020194155)—note that the full PRIMSA checklist can be found in Supplementary Table 1 and justifications of any deviations from the registered protocol can be found in Supplementary Table 2.

### Search Strategy

Databases were searched from 1/12/2019 to 19/4/2021 including Embase, MEDLINE, Pubmed, Scopus, Web of Science, CINAHL, The Cochrane library UK clinical Research Network: Portfolio database, and the International Standard Registered Clinical/soCial sTudy Number (ISRCTN) registry, using the following search terms:

(SARSCoV-2 OR 2019-nCoV OR COVID-19 OR coronavirus OR “Wuhan Coronavirus”)
AND
(2019 or 2020)
AND
(asthma^*^ OR COPD OR “chronic obstructive pulmonary disease”)

No other limiters were applied.

### Study Selection

Two researchers (MV,SW) independently screened titles and abstracts of all identified studies after duplicates were removed. Discrepancies between reviewers were resolved by discussion before screening full texts independently against the inclusion criteria. If it was not possible to determine whether a study met the inclusion criteria from the title and/or abstract, it was marked for a full paper review. Where necessary, the reviewers contacted corresponding authors to request missing information or clarification. All references were imported to Mendeley.

### Study Inclusion and Exclusion

Two reviewers (MV & SW) independently screened all titles and abstracts. The relevance of each study was assessed according to the inclusion and exclusion criteria. Studies were included if they met the following criteria.

#### Population

Studies including humans with COPD and/or asthma and a confirmed case (via polymerase chain reaction or antibody test) of COVID-19 were included in this review. Children <18 yrs and animal studies were excluded from this review. We also excluded studies on previous human coronaviruses: 229E, NL63, OC43, HKU1, MERS-CoV, and SARS-CoV.

#### Intervention

Observational studies, including case-control and cohort studies were included. Randomized studies that reported the prognostic role of asthma/COPD in *post-hoc* analyses (e.g., Cox regression models) were also included.

#### Comparison

Comparator groups include humans with confirmed COVID-19 and no evidence of COPD and/or asthma.

#### Outcomes

Studies had to report one or more of the following:

1. Number of COVID-19 cases hospitalised *vs*. COVID-19 cases non-hospitalised cases.
2. Number of hospitalised COVID-19 cases treated in intensive care unit (ICU) *vs*. hospitalsation but not admitted for ICU care.
3. Number of COVID-19 related deaths *vs*. survival.

Furthermore, studies were excluded if they were:

1. Not written in English.
2. Not peer reviewed (e.g., preprints).
3. Studies in a non-adult (<18 years) population.
4. Had insufficient data to calculate an adjusted odds ratio (aOR; adjusted for more than one COVID-19 related covariate) related to the stated outcomes.

### Data Extraction

Data was extracted by two reviewers (MT & MV) and included: first author, study title, date of study, dates in which study data were collected, country, aim/objective, study type, number of participants, disease investigated, method of disease diagnoses, method of COVID-19 diagnosis, outcome type, sample size, participant characteristics, adjusted OR and 95% confidence intervals (CIs) (or raw data in which an adjusted odds ratio could be calculated), details of confounding variables the OR was adjusted for. Where data was missing, required clarification or particular variables of interest were not reported in the paper, corresponding authors were contacted to enable inclusion in the meta-analysis, and given 2 weeks to respond. If no response was received within 2 weeks, or the data was unavailable, these studies were excluded.

### Quality Assessment

Risk of bias was assessed by two independent researchers (MT & MV) using the Quality In Prognosis Studies (QUIPS) tool (27). The QUIPS is a non-scoring appraisal tool for assessing the scientific validity of articles, which requires the identification of whether or not relevant information is present in each article using a yes, no or not applicable rating, with an overall verdict of “low,” “medium,” or “high” risk of bias. Any discrepancies over the final risk of bias verdict was made by consensus, with involvement of a third review author (SP) where necessary.

### Statistical Analysis

Due to anticipated heterogeneity, a random-effects model was conducted using the DerSimonian and Laird method, with studies weighted according the inverse variance, using Comprehensive Meta-Analysis (28). The meta-analysis was conducted using the following steps:

1. Adjusted odds ratios (aORs), or adjusted Hazard Ratios (aHRs) and 95% CIs were inputted (with significance set as *p* = 0.05). Note that if the raw data were available, a binary logistic regression was conducted.
2. Heterogeneity between studies was assessed using the *I*^2^ statistic (29). If high (>50%) heterogeneity was found, sub-group analyses were conducted based on total participants (>10 vs. <10k participants).
3. Publication bias was assessed with a visual inspection of funnel plots and with the Egger bias test (30). As per the recommendations by Fu et al. (31) and Sterne et al. (32), these tests were only conducted if the number of studies in each analysis exceeded ten.
4. Sensitivity analyses were conducted to assess the robustness of the pooled effect sizes through the one study removed method.

### Certainty of Evidence

To ascertain the certainty of the evidence, the Grading of Recommendations, Assessment, Development and Evaluations (33) (GRADE) framework was used.

## Results

**Figure 1:**
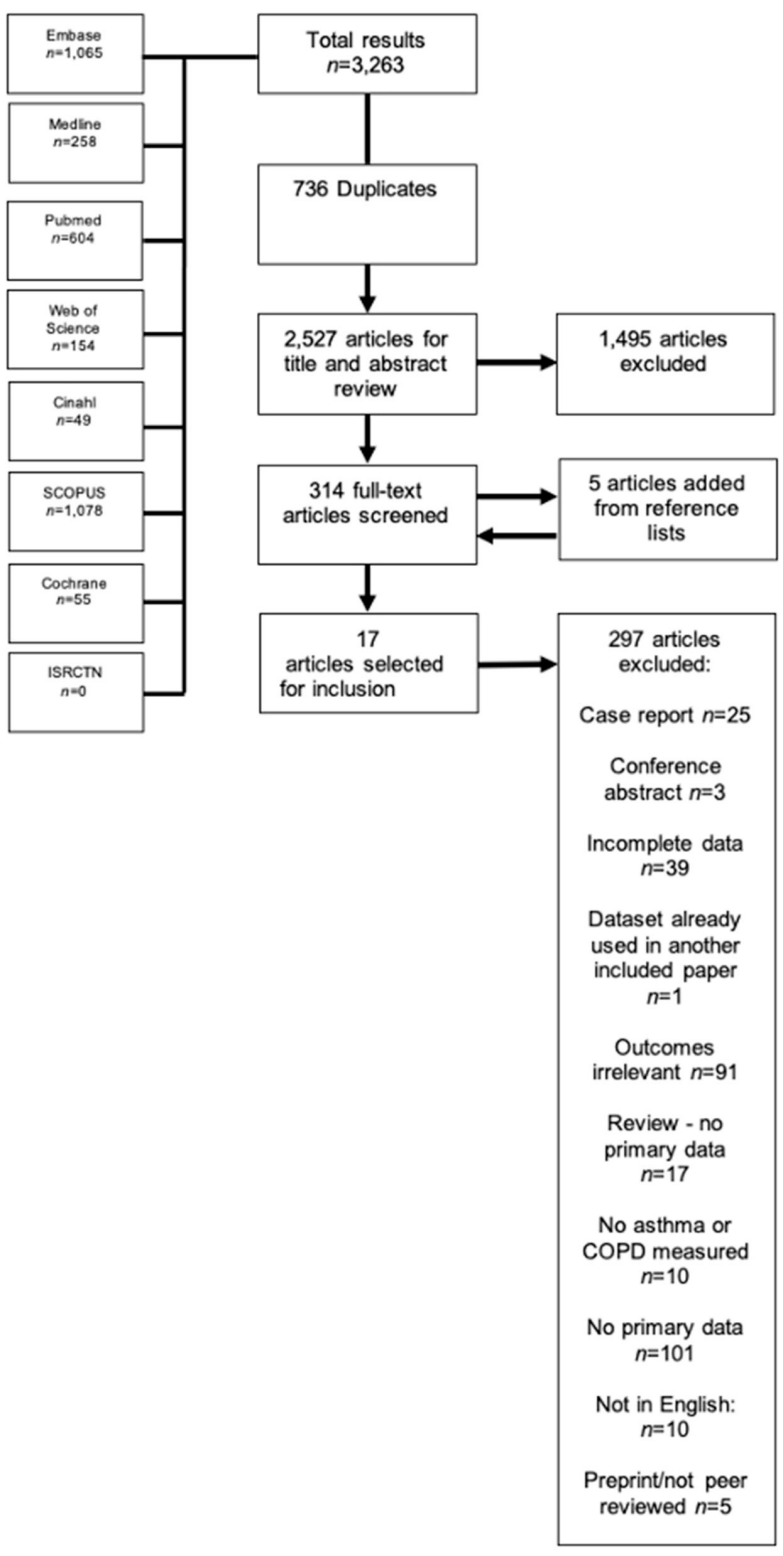
PRISMA flowchart of included studies.

The literature search yielded 3,701 results, of which 780 were duplicates and were automatically removed, leaving 2,921 studies to be screened using the title and abstract. Of these studies, 416 full-texts were screened, where five extra studies were obtained by way of reference lists, resulting in 421 full texts that were finally screened. Thirty-eight studies appeared to be eligible for inclusion, however one (34) was excluded because the reported 95% CIs were not symmetrical, and therefore could not be pooled, leaving 37 finally eligible for inclusion (21, 35–69). The full PRISMA flowchart is shown in Figure 1, and a full list of excluded studies with reasons for exclusion can be found in Supplementary Table 2. There were a total of 1,678,992 participants across the included studies, with a mean age range of 45.7–81.9 years. Of the included studies, 10 (38, 42, 46, 48, 51, 55, 56, 58, 66, 69) examined outcomes in both asthma and COPD, seven (21, 43, 50, 52, 63, 64) examined outcomes exclusively in asthma, and the remaining 20 studies (37, 39–41, 44, 45, 47, 49, 53, 57, 59–62, 65, 67, 68, 70, 71) reported on outcomes exclusively regarding COPD. All but one study was classified as having low risk of bias (see Supplementary Table 4 for full QUIPS scoring). Full descriptive characteristics of included studies are shown in Table 1.

**Table 1:** Descriptive characteristics of included studies.

**Authors**	**Study design**	**Country**	**Total *n***	**Age (mean)**	**Percentage female**	**Type of outcome(s) measured**	**Disease**	**Method of asthma diagnosis**	**Method of COPD diagnosis**	**Confounding/adjusted variables**	**Conflict of interest**	**Risk of bias**
Atkins et al. (38)	Cohort	UK	268,704	73.1	NR	Hospitalsation risk; mortality risk	Asthma or COPD	“existing diagnoses were available from baseline questionnaires (2006–2010) eliciting participant reports of doctor-diagnosed disease. New disease diagnoses since baseline were from linked electronic medical records to hospital inpatient routine data (to March 2017), coded according to the International Classification of Diseases 10th revision (ICD-10)”		Age group, sex, ethnicity, education, baseline assessment centre, CHD, Atrial fibrillation, stroke, hypertension, T2D, CKD, depression, dementia, asthma, COPD, Osteoporosis, previous delirium, previous pneumonia, previous falls/fragility fractures.	Reported—none declared	Low
Attaway et al. (39)	Cohort	USA	2527	NR	NR	Hospitalsation risk; ICU admission risk; mortality risk	COPD	–	“Patients were asked if they had a diagnosis of COPD, and the diagnosis was confirmed if it was also included in the patient's medical chart”	Age, race, sex, BMI, smoking status (current vs. former), hypertension, cancer, diabetes mellitus, coronary artery disease, immunosuppressive therapy.	Reported—none declared	Low
Aveyard et al. (55)	Retrospective cohort	UK	811	NR	NR	Mortality risk	Asthma and COPD	NR	NR	Age, sex, ethnicity, socioeconomic status, region of England, body-mass index (categorical variable), and smoking status (with current intensity of smoking as categorical variables), on-smoking-related illness (hypertension, type 1 diabetes, chronic liver disease, chronic neurological disease) and smoking-related illness (coronary heart disease, stroke, atrial fibrillation, type 2 diabetes, chronic kidney disease).	Reported—several potential conflicts declared	Low
Azoulay et al. (59)	Retrospective cohort	France	376	NR	NR	Mortality risk	COPD	–	NR	Age, comorbidities (asthma, diabetes, COPD, hypertension, immunosuppression), time from viral symptom onset to ICU admission, acute kidney injury, and troponin	Reported—none declared	Low
Bloom et al. (69)	Retrospective cohort	UK	47,398	NR	NR	Mortality risk	Asthma and COPD	NR	NR	Age, sex, ethnicity, smoking, obesity, malignancy, chronic cardiac disease, CKD, and centre	Reported—several potential conflicts declared	Low
Cellina et al. (40)	Retrospective observational	Italy	246	63.0	31.0%	Mortality risk	COPD	–	NR	Age, diabetes, and radiological outcomes	Reported—none declared	Low
Choi et al. (21)	Cohort	Korea	7,590	NR	NR	ICU admission risk; mortality risk	Asthma	“An asthma diagnosis was determined when patients visited the hospital (at least once) due to asthma symptoms from January 2019 to December 2019. Furthermore, only patients who met the following criteria during the assessment period were regarded as having asthma: (1) ICD- 10 codes for asthma (J45 and J46) as primary diagnosis or first sub-diagnosis; and (2) prescription of asthma medications on at least 2 occasions during outpatient visits or prescription of asthma medication following an outpatient visit and admission with treatment using systemic corticosteroids during the assessment period.”	–	Age, sex, and underlying conditions	Reported—none declared	Low
Choi et al. (54)	Retrospective cohort	South Korea	4,057	NR	60.4%	Mortality risk	Asthma	NR	–	Age, sex, obesity, systolic blood pressure, diastolic blood pressure, heart rate, temperature, diabetes, hypertension, heart failure, chronic heart disease, chronic obstructive pulmonary disease, chronic kidney disease, cancer, chronic liver disease, rheumatic or autoimmune disease, and dementia.	Reported—none declared	Low
De Vito et al. (41)	Retrospective observational	Italy	87	72 (median)	35.6%	Mortality risk	COPD	–	NR	Age >72 years, Hypertension, > 3 comorbidities, >5 comorbidities, non-compliance, moderate ARDS, lymphocyte <900/mm^3^	Reported—none declared	Low
De Vito et al. (57)	Retrospective cohort	Italy	264	81.9 (10.1)	62.5%	Mortality risk	COPD	–	NR	Age, sex, hypertension, diabetes, neurological syndrome, hypokinetic disease, autonomy, fever + dyspnoea, LMWH	Reported—none declared	Low
Giannouchos et al. (42)	Cross-sectional	Mexico	89,756	46.2	43.6%	Hospitalsation risk; ICU admission risk	Asthma and COPD	NR	NR	Age, gender, smoking, CKD, diabetes, immunosuppression, obesity, hypertension, CVD, asthma or COPD	Reported—none declared	Low
Girardin et al. (56)	Retrospective cohort	USA	4,446	NR	NR	Mortality risk	Asthma and COPD	NR	COPD was defined as presence of chronic bronchitis or emphysema.	Age, sex, PAD, low income, asthma, ethnicity, obesity, CAD, cancer, smoking, diabetes, auto-immune disease, hyperlipidaemia, sleep apnoea, hypertension	Reported—none declared	Low
Grandbastien et al. (43)	Cross-sectional	France	106	63.5 (median)	37.7%	ICU admission ris	Asthma	“clinical diagnosis of asthma based on the clinical history recorded by medical staff”	–	Age, sex, hypertension, diabetes, body mass index <30, and heart failure	Reported—one author reports conflict of interest with pharmaceutical companies	Low
Grasselli et al. (60)	Retrospective cohort	Italy	3,988	NR	20.1%	Mortality risk	COPD	- -	NR	Age, sex, respiratory support type, HTN, hypercholesterolemia, heart disease, T2D, malignancy, ACE inhibitor therapy, ARB therapy, statin, diuretic, PEEP at admission, Fio2 at admission, Pao2/Fio2 at admission	Reported—several potential conflicts declared	Low
Guan et al. (66)	Retrospective cohort	China	39,420	55.7 (NR)	NR	Mortality risk	Asthma and COPD	NR	NR	Age, sex, other systemic comorbidities	Reported—none declared	Low
Gupta et al. (44)	Cohort	USA	2,215	60.5	35.2%	Mortality risk	COPD	–	“Per chart review”	Age, sex, race, hypertension, diabetes, body mass index, coronary artery disease, congestive heart failure, current smoking status, active cancer, duration of symptoms before ICU admission, and covariates assessed at ICU admission (lymphocyte count, ratio of the PaO2 to the fraction of inspired oxygen [FIO2], shock, and the kidney, liver, and coagulation components of the sequential organ failure assessment score).	Reported—several authors report conflict of interest	Low
Harrison et al. (45)	Retrospective cohort	USA	31,461	50 (median)	54.5%	Mortality risk	COPD	–	NR	Age, sex, ethnicity, myocardial infarction, congestive heart failure, peripheral vascular disease, cerebrovascular disease, dementia, rheumatic disease, peptic ulcer disease, mild liver disease, moderate/severe liver disease, diabetes, hemiplegia or paraplegia, renal disease, any malignancy, metastatic solid tumor, AIDS/HIV	Reported—several authors report conflict of interest	Low
Hernandez-Galdamez et al. (46)	Cross-sectional	Mexico	211,003	45.7	45.3%	Hospitalsation risk; ICU admission risk; mortality risk	Asthma and COPD	“The information is obtained through a dichotomous questionnaire that the physician fills with the information provided by the patient.”		Age, sex, CKD, immunosuppression, diabetes, hypertension, cardiovascular disease, COPD or asthma, obesity and smoking.	Reported—none declared	Low
Ho et al. (64)	Retrospective cohort	USA	10,523	58.35 (18.81)	45.8%	Hospitalsation risk; ICU admission risk; mortality risk	Asthma	NR		Age, sex, BMI, race, COVID-19 disease severity, Charleston Comorbidity Index, COPD, C-reactive protein (>150 mg/L), interleukin-6 (>80 mg/L), ferritin (>2,000 ng/L), D-dimer (>2.0 mg/L), use of anticoagulation, use of corticosteroids, and smoking (current and former).	Reported—none declared	Low
Hu et al. (47)	Cohort	China	821	NR	NR	Mortality risk	COPD	–	“COPD patients diagnosed by lung function”	Age, sex, hypertension, diabetes, CAD, CVD, Malignancy, CKD, chronic liver disease	Reported—none declared	Low
Hu et al. (72)	Retrospective cohort	China	213	44 (median)	NR	ICU admission risk	COPD	–	NR	Age, Dyspnoea, Poor appetite, WBC>10 ×10-9/l, D-dimer>0.5 mg/l, Albumin <35 g/L, ALT, AST, LDH.	Reported—none declared	Low
Jiang et al. (68)	Retrospective cohort	China	281	NR	NR	Mortality risk	COPD	–	NR	Age, sex, anorexia, comorbidities, CD8+ count, lymphocyte count, CRP, D-dimer, LDH, high sensitivity troponin I, osmotic pressure, PCT, and SOFA score on ICU admission	Reported—none declared	Low
Kammar-Garcia et al. (51)	Cohort	Mexico	13,842	NR	NR	Hospitalsation risk; ICU admission risk; mortality risk	Asthma and COPD	“Self-report and defined as present or absent”	Age, sex, pneumonia, diabetes, asthma or COPD, immunosuppression, hypertension, CVD, obesity, CKD, other comorbidities	Not reported	Medium	Low
Lee et al. (67)	Retrospective cohort	South Korea	4,610	NR	NR	Mortality risk	COPD	–	Medical records—Identification of COPD patients with ICD-10 codes (J43 and J44 except J43.0)	Age, sex, and Charleston Comorbidity Index score	Reported—none declared	Low
Li et al. (53)	Case-series	China	204	68 (median)	51%	Mortality risk	COPD	–	NR	None	Reported—none declared	Low
Mahdavinia et al. (52)	Case-series	USA	1,003	NR	NR	Hospitalsation risk; mortality risk	Asthma	“asthma diagnosis based on Global Initiative for Asthma (GINA) guidelines”	-	None	Reported—none declared	Low
Martos-Benitez et al. (37)	Retrospective cohort	Mexico	38,324	46.9 (15.7)	41.7%	ICU admission risk; mortality risk	COPD	–	NR	Age, sex, smoking habit, time from symptoms onset to medical contact, and all the comorbidities	Reported—none declared	Low
Murillo-Zamora et al. (58)	Retrospective cohort	Mexico	66,123	NR	NR	Mortality risk	Asthma and COPD	NR	NR	Age, sex, diagnosed pneumonia at admission, tobacco use, obesity, COPD, diabetes, arterial hypertension, immunosuppression, CKD	Reported—none declared	Low
Parra-Bracamonte et al. (48)	Cohort	Mexico	331,298	44 (median)	46.2%	Mortality risk	Asthma and COPD	As confirmed by dataset used—no specific method reported		Age, sex, smoking status, hospitalsation, pneumonia, hypertension, obesity, diabetes, cardiopathy, COPD or asthma, immunosuppressed, CKD, other complications.	Not reported	Low
Rosenthal et al. (63)	Retrospective cohort	USA	727	49.46 (17.93)	NR	Hospitalsation risk	Asthma	NR	–	Age, BMI, race, and a number of comorbidities (chronic kidney disease, coronary artery disease or congestive heart failure, diabetes, and hypertension)	Reported—none declared	Low
Timerlake et al. (65)	Retrospective cohort	USA	274	NR	NR	ICU admission risk; mortality risk	COPD	–	NR	Age, sex, race, admission diagnosis (COVID-19 vs. other), CAD, and obesity	Reported—several potential conflicts declared	Low
Wang et al. (61)	Case-series	China	339	69 (median)	51.0%	Mortality risk	COPD	–	NR	Age, CVD, cerebrovascular disease	Reported—none declared	Low
Wang et al. (62)	Retrospective cohort	China	141	64 (median)	30.0%	Mortality risk	COPD	–	NR	Ventilation status, creatinine ?104 umol/; vs. <104 umol/l and chronic renal diseases	Reported—none declared	Low
Wang et al. (70)	Case-series	USA	1,827	54 (median)	67.4%	Hospitalsation risk; ICU admission risk; mortality risk	COPD	-	NR	Age, sex, race, marital status, educational level, insurance type, smoking history, BMI, diabetes, CKD, CLD, CVD, HTN, allergic rhinitis	Reported—several potential conflicts declared	Low
Wu et al. (49)	Retrospective observational	China	443	NR	NR	ICU admission risk	COPD	–	NR	Age, sex, smoking status, diabetes, hypertension, coronary heart disease, cerebrovascular diseases, hepatitis B infection, cancer, chronic renal diseases, immunodeficiency.	Reported—none declared	Low
Yoshida et al. (71)	Case-series	USA	776	60.5 (16.1)	NR	ICU admission risk; mortality risk	COPD	–	NR	Age, sex, hospital site, and the Charleston Comorbidity Index	Reported—none declared	Low
Zhu et al. (50)	Cohort	UK	492,768	NR	NR	Hospitalsation risk	Asthma	Measurement of genetic asthma phenotypes	-	Age, sex, race/ethnicity, and BMI	Reported—none declared	Low

### Meta-Analysis

#### Risk of COVID-19 Related Hospitalsation

**Figure 2:**
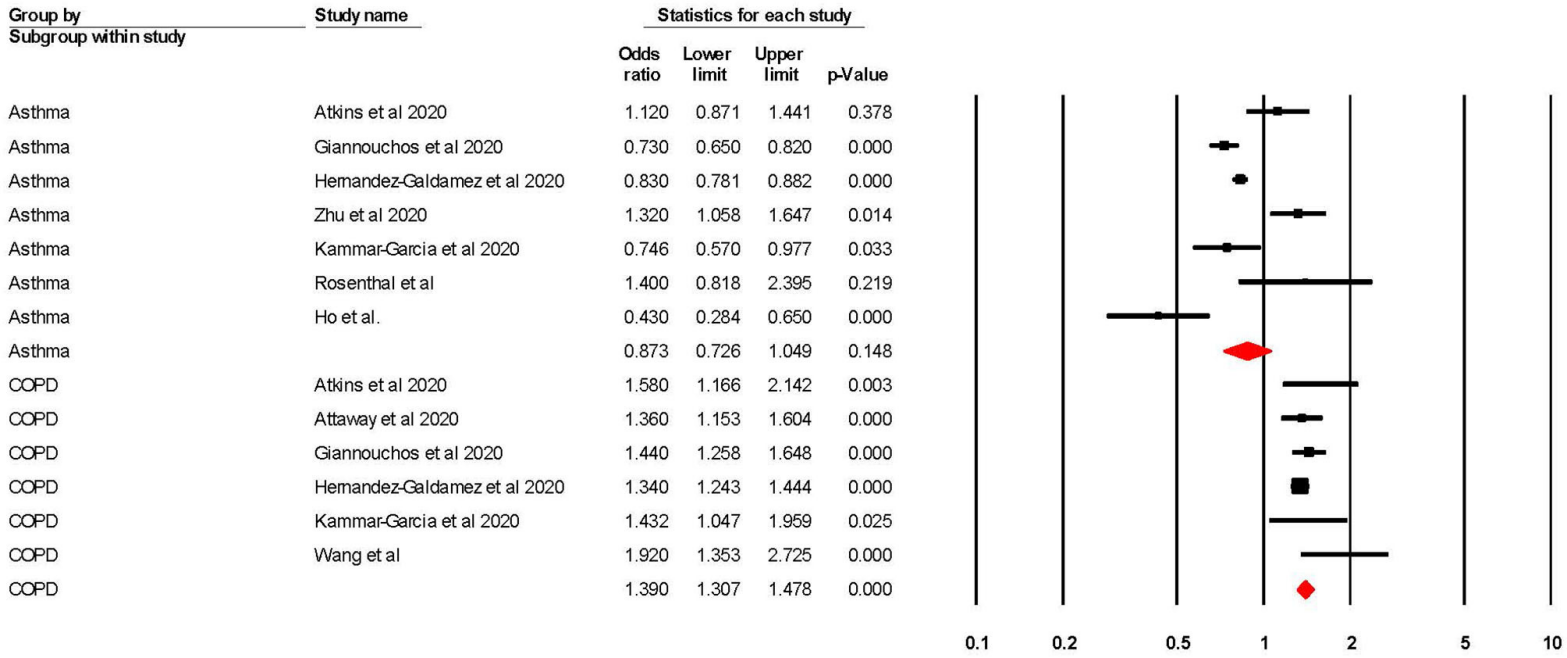
Forest plot showing odds ratios (adjusted for at least one confounder) for COVID-19 related hospitalisation in subjects with asthma or Chronic Obstructive Pulmonary Disease (COPD).

When adjusted for one or more comorbidity, the pooled aOR was 0.87 (95% CI 0.73–1.05; *p* = 0.15; *I*^2^ = 85.36) for asthma and 1.39 (95% CI 1.31–1.48; *p* = < 0.001; *I*^2^ = 4.24) for COPD (see Table 2 and Figure 2). The sensitivity analysis found that the removal of any one study did not significantly change the direction of results for either asthma or COPD (see Supplementary Figures 1, 2 for full details).

**Table 2:** Meta-analysis showing the pooled adjusted risk of unfavorable COVID-19 outcomes in subjects with asthma or COPD.

**Study details**			**Meta-analysis**		**Heterogeneity**	**Publication bias**	**GRADE rating**
**Lung disease**	**Number of studies**	**Number of participants**	**Odds ratio (95% CI)**	***p*-value**	***I*^**2**^**	**Egger bias and *p*-value**	
**Hospitalisation**							
Asthma	7	1,087,689	0.873 (0.726–1.049)	0.148	85.355	0.747*p* = 0.678	Moderate (downgraded due to high heterogeneity)
COPD	6	588,025	1.390 (1.307–1.478)	<0.001	4.236	1.453*p* = 0.050	Moderate (downgraded due to possible publication bias)
**ICU admission**							
Asthma	4	167,849	0.746 (0.545–1.020)	0.067	87.198	−1.979*p* = 0.653	Moderate (downgraded due to high heterogeneity)
COPD	9	197,108	1.336 (1.139–1.566)	<0.001	66.643	1.537*p* = 0.075	Moderate (downgraded due to high heterogeneity)
**Mortality (aORs)**							
Asthma	7	876,759	0.827 (0.711–0.961)	0.013	61.481	0.007*p* = 0.996	Moderate (downgraded due to high heterogeneity)
COPD	17	950,502	1.276 (1.176–1.385)	<0.001	34.508	0.881*p* = 0.038	Moderate (downgraded due to possible publication bias)
**Mortality (aHRs from Cox regression models)**							
Asthma	4 (5 outcomes)	122,786	0.930 (0.865–1.000)	0.049	64.176	1.400*p* = 0.414	Moderate (downgraded due to high heterogeneity)
COPD	8 (9 outcomes)	123,886	1.296 (1.170–1.436)	<0.001	88.386	2.179*p* = 0.093	Moderate (downgraded due to high heterogeneity)

*GRADE, Grading of Recommendations, Assessment, Development, and Evaluations; COPD, Chronic Obstructive Pulmonary Disease; aOR, adjusted odds ratio; aHR, adjusted hazard ratio*.

#### Risk of COVID-19 Related ICU Admission

**Figure 3:**
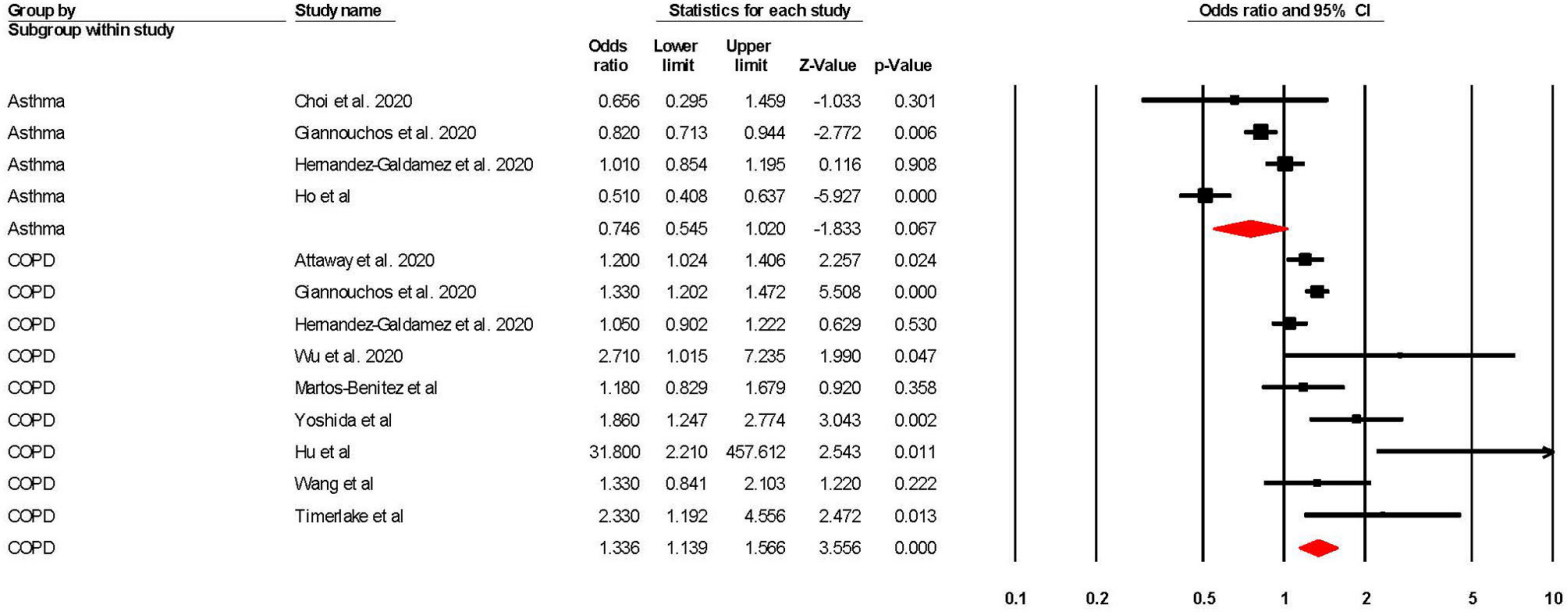
Forest plot showing odds ratios (adjusted for at least one comorbidity) for COVID-19 related intensive care admission in subjects with asthma or Chronic Obstructive Pulmonary Disease (COPD).

When adjusted for one or more comorbidity, the pooled aOR was 0.75 (95% CI 0.55–1.02; *p* = 0.07; *I*^2^ = 87.20) for asthma and 1.34 (95% CI 1.14–1.57; *p* = <0.001; *I*^2^ = 66.64) for COPD (see Table 2 and Figure 3). The sensitivity analysis found that for asthma the aOR became significant with the removal of one study (46) (OR = 0.65 95% CI 0.44–0.97 *p* = 0.04). The removal of any one study did not significantly change the direction of results for COPD (see Supplementary Figures 3, 4 for full details).

#### Risk of COVID-19 Related Mortality

**Figure 4:**
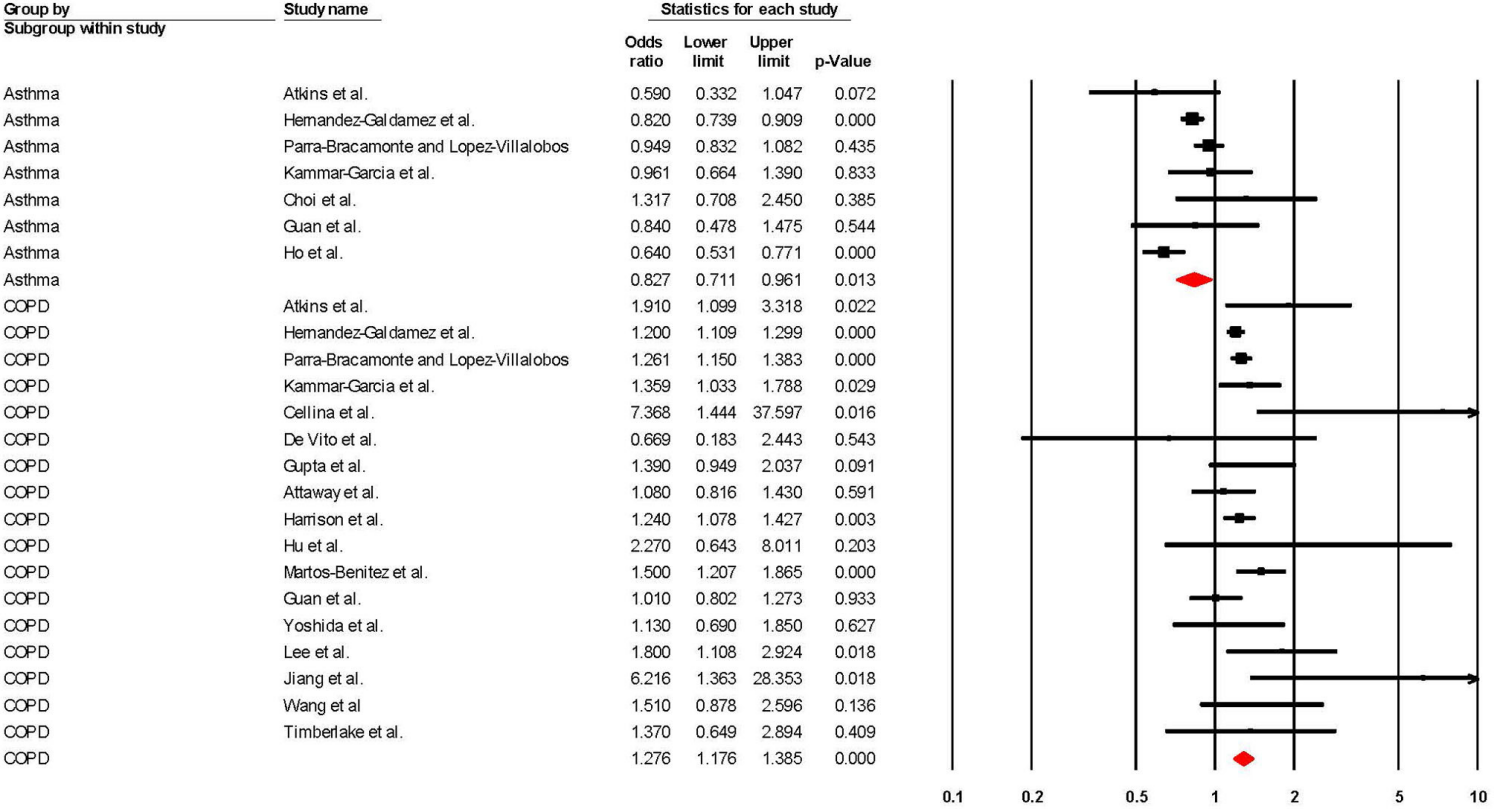
Forest plot showing odds ratios (adjusted for at least one comorbidity) for COVID-19 related overall mortality in subjects with asthma or Chronic Obstructive Pulmonary Disease (COPD).

When adjusted for one or more comorbidity, the pooled aOR was 0.83 (95% CI 0.71–0.96; *p* = 0.01; *I*^2^ = 61.48) for asthma and 1.28 (95% CI 1.18–1.39; *p* = < 0.001; *I*^2^ = 34.51) for COPD (see Table 2 and Figure 4). The sensitivity analysis found that for asthma the aOR became non-significant with the removal of one study (46) (OR = 0.83 95% CI 0.66–1.05 *p* = 0.118), and the results did not significantly change for COPD when any one study was removed (see Supplementary Figures 5, 6 for full details).

**Figure 5:**
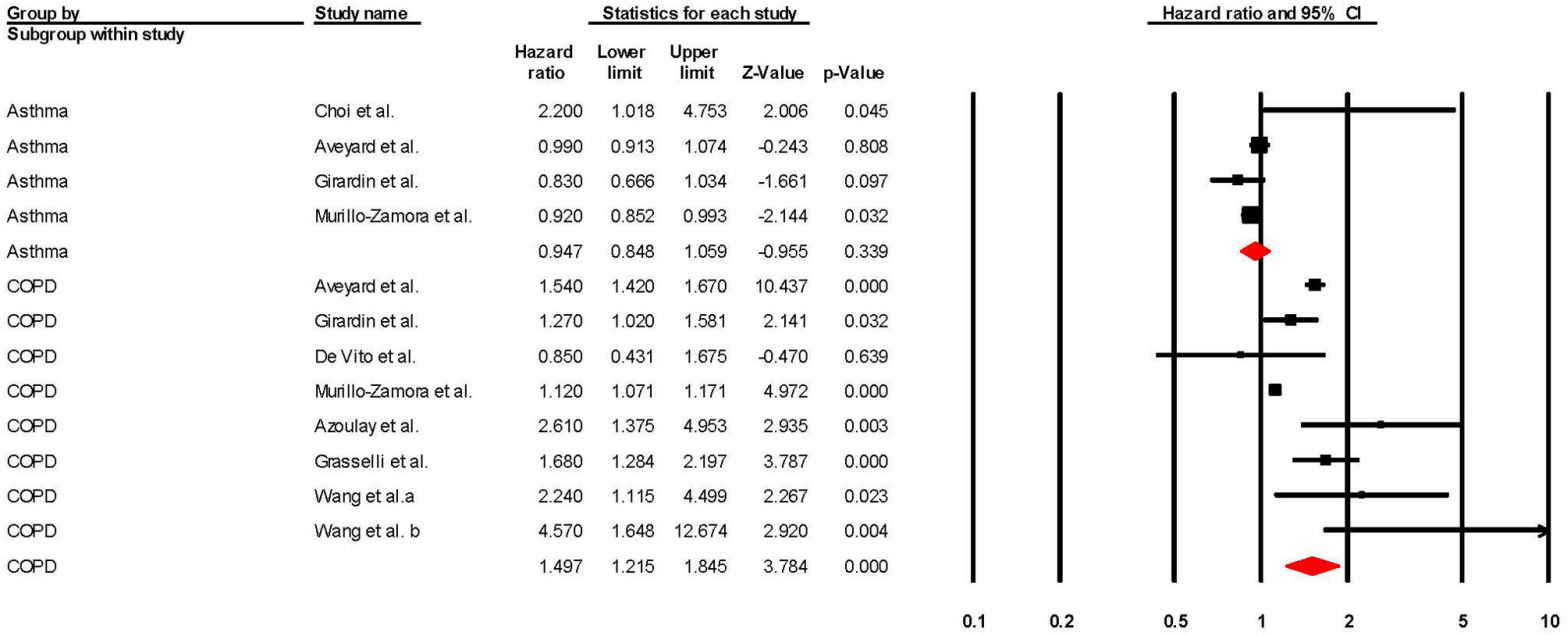
Forest plot showing Cox regression hazard ratios (adjusted for at least one comorbidity) for COVID-19 related overall mortality in subjects with asthma or Chronic Obstructive Pulmonary Disease (COPD).

Regarding studies that reported aHRs in the form of Cox regression models, the pooled risk of mortality was 0.93 (95% CI 0.87–1.00; *p* = 0.049; *I*^2^ = 64.18) for asthma and 1.30 (95% CI 1.17–1.44; *p* = <0.001; *I*^2^ = 88.39) for COPD (see Table 2 and Figure 5). The sensitivity analysis found that the removal of any one study did not significantly change the direction of results for COPD, and the removal of any one of three studies (56, 58, 69) changed the significance of results in asthma (see Supplementary Figures 7, 8 for full details).

##### Certainty of Evidence Using the GRADE Approach

Using the GRADE (33) approach, all of the results were rated as being a “moderate” level of certainty. The two reasons why the level of evidence was not rated as “high” was because of either (1) high heterogeneity, or (2) the presence of publication bias.

##### Sub-Group Analyses

When sub-grouped between studies with >10 vs. <10k participants, no significant changes were found, except for in risk of mortality (as measured by Cox regression) in participants with COPD. It was found that studies with >10k participants yielded significantly lower (*p* = 0.001) risk of mortality (aHR = 1.13 95% CI 1.10–1.17) when compared to studies that had <10k participants (aHR = 1.59 95% CI 1.31–1.94), and also yielded lower heterogeneity in this subgroup (>10k = 36.19%; <10k = 58.32%). Although the differences between sub-groups were significant, both pooled aHRs were still, respectively, statistically significant. Full information can be found in Table 3 and in Supplementary Figures 9–16.

**Table 3:** Sub-group analyses showing the pooled adjusted risk of unfavorable COVID-19 outcomes in participants with asthma or COPD stratified >10 vs. <10k participants.

**Study details**			**Meta-analysis**			**Heterogeneity**
**Lung disease**	**Sub-group**	**Number of studies**	**Odds ratio (95% CI)**	***p*-value**	**Differences between groups**	***I*^**2**^**
**Hospitalisation**						
Asthma	>10k	1	1.400 (0.818–2.395)	0.219	*p* = 0.079	0.000
	<10k	6	0.841 (0.697–1.014)	0.070		86.609
COPD	>10k	4	1.374 (1.291–1.463)	<0.001	*p* = 0.463	0.000
	<10k	2	1.559 (1.120–2.169)	0.008		67.174
**ICU admission**						
Asthma	>10k	3	0.757 (0.537–1.065)	0.110	*p* = 0.748	91.376
	<10k	1	0.656 (0.295–1.459)	0.301		0.000
COPD	>10k	3	1.191 (0.994–1.426)	0.058	*p* = 0.077	69.159
	<10k	6	1.708 (1.196–2.441)	0.003		65.159
**Mortality (aORs)**						
Asthma	>10k	6	0.808 (0.695–0.938)	0.013	*p* = 0.133	62.813
	<10k	1	1.317 (0.708–2.450)	0.005		0.000
COPD	>10k	7	1.251 (1.160–1.349)	<0.001	*p* = 0.320	37.046
	<10k	10	1.425 (1.115–1.821)	0.005		36.935
**Mortality (aHRs from Cox regression models)**						
Asthma	>10k	2 (3 outcomes)	0.913 (0.852–0.978)	0.009	*p* = 0.529	59.036
	<10k	3	0.993 (0.772–1.275)	0.954		69.146
COPD	>10k	2 (3 outcomes)	1.132 (1.097–1.168)	<0.001	*p* = 0.001	36.191
	<10k	7	1.590 (1.305–1.937)	<0.001		58.320

*COPD, Chronic Obstructive Pulmonary Disease; aOR, adjusted odds ratio; aHR, adjusted hazard ratio*.

## Discussion

This meta-analysis included 37 studies examined the adjusted risks of COVID-19 related hospitalsation, ICU-admission, and mortality in populations with and without either asthma or COPD. The analysis suggests with a moderate level of certainty that COPD is a significant risk factor for COVID-19 related hospitalsation, ICU admission, or mortality when the risks were adjusted for at least one comorbidity. Furthermore, with a moderate level of certainty, asthma was not shown to be a significant risk factor for COVID-19 related hospitalsation, ICU admission, or mortality when adjusted for at least one comorbidity.

COPD was shown to be a significant risk factor in all three outcomes, with the sensitivity analysis reporting robustness in all outcomes. These results broadly agree with previous meta-analyses exploring similar outcomes in this population (10–14). When directly comparing reported risks, this study shows a marked decrease in mortality risk (5.69 vs. 1.25) when compared to Lippi and Henry (10), which would be expected. Although the mechanisms that underpin this risk are not clear, several hypotheses, including the increased expression of the angiotensin-converting enzyme 2 (ACE-2) in COPD patients, have been reported as COVID-19's route of entry into susceptible cells (73). Furthermore, it has been reported that morbidity and mortality in COPD patients are frequently related to acute exacerbation (12), and severe respiratory failure (67) which may add to already compromised respiratory capacity among COVID-19 patients (12, 74, 75). Moreover, the effect of smoking could be a reason why people with COPD appear to have increased COVID-19 risks; indeed, a recent systematic review and meta-analysis (76) reported that both current and former smokers have increased risks of COVID-19 related deaths, although these risks do not appear to have been adjusted for any co-variates. Further exploration into adjusted smoking risk, in particular adjusted for COPD and/or asthma presence, would be beneficial.

Other comorbidities have also been shown to be significant risk factors for unfavorable COVID-19 related outcomes including (but not limited to), hypertension (4), diabetes (5), and obesity (3). It is difficult to directly compare our results with previous data as these previous estimates report unadjusted data making true risks of each comorbidity hard to compare. We agree with Jordan et al. (20) and recommend that future studies aim to report risks based on adjustments for, at the very least, age, sex, and smoking status so that true risks can be determined. It is recommended that clinicians continue to consider COPD patients to be at greater risk of COVID-19 related morbid outcomes. Individuals with COPD should take extra precautions to ensure that exposure to COVID-19 is minimal.

Although asthma has been related to worse outcomes in other viral infections, including other forms of coronavirus (16, 17), our analysis did not suggest asthma as a significant risk factor for any of the outcomes measured in this review, apart from mortality (measured as a non-time dependent OR), however sensitivity analysis suggested that the significance of this outcome was subject to the influence of one large study. These results broadly agree with previous meta-analyses that concluded that asthma was not a significant risk factor for either mortality or “severe” health outcomes (14, 18, 35, 77). When directly comparing reported risks across these meta-analyses, this study's mortality risk is lower (0.83 and 0.93 vs. 0.96 and 1.03) (35, 77), which is an expected result given we pooled adjusted ORs and the other meta-analyses were not adjusted for any other covariates. These results, however, need to be interpreted with caution as the included studies have used asthma as an umbrella term and did not differentiate between different types or severities of the disease. The National Health Service (NHS) in the UK has severe asthma listed “high risk of severe outcomes,” and other severities at “moderate risk” of COVID-19 (78), and although this study does not support this, more data is required to differentiate between different severity of asthma, and, as such, individuals with asthma should still aim to minimize their risk of COVID-19 exposure.

Although this is the first review to systematically examine risks of unfavorable COVID-19 outcomes in populations with asthma or COPD with effect sizes adjusted for at least one covariate, our results should be considered within its limitations. Firstly, although the majority were deemed as low risk of bias, the effect of methodological bias cannot be ruled out. Secondly, the pooling of adjusted ORs (with different studies adjusting for different covariates) inherently creates a degree of inconsistency, meaning that the results should be treated only as indicative. Thirdly, there was considerable heterogeneity in some of the reported analyses, especially in the asthmatic populations, which could not be explained by the presence of large studies vs. smaller ones. One probable reason for this is the different asthma diagnosis methods, in particular regarding the type and severity of asthma. Furthermore, there was some evidence of publication bias, which could not be explained. Lastly, meta-analyses have inherent limitations: their findings are dependent on estimates selected from each primary study and thus are dependent on the accuracy of primary studies (79).

## Conclusions

COPD is significantly associated with worse COVID-19 related, hospital admission, ICU admission and mortality, even when adjusted for at least one comorbidity. Asthma, when pooling risks were adjusted for other comorbidities, was not associated with a higher risk of COVID-19 related hospitalsation, ICU admission and mortality. Clinicians should note these findings when dealing with patients with these comorbidities. Furthermore, individuals with COPD should take special precautions to limit the risk of COVID-19 exposure to negate these potential outcomes.

## Data Availability Statement

The original contributions presented in the study are included in the article/Supplementary Material, further inquiries can be directed to the corresponding author/s.

## Author Contributions

MT and MV acquisition and analysis. MT, MV, and SP drafted the work. MT and MV verified the underlying data. All authors made substantial contributions to the conception, design of the work, interpretation of data for the work, revising it critically for important intellectual content and final approval of the version to be published, and agreement to be accountable for all aspects of the work in ensuring that questions related to the accuracy or integrity of any part of the work are appropriately investigated and resolved.

## Conflict of Interest

The authors declare that the research was conducted in the absence of any commercial or financial relationships that could be construed as a potential conflict of interest.
