# Supplementary material for: The Risk of COVID-19 Related Hospitalsation, Intensive Care Unit Admission and Mortality in People With Underlying Asthma or COPD: A Systematic Review and Meta-Analysis
Source: Front Med (Lausanne). 2021 Jun 16;8:668808. doi: 10.3389/fmed.2021.668808 (PMC8242585; doi:10.3389/fmed.2021.668808)
Supplement: Supplementary file 1 [file Data_Sheet_1.docx]

Supplementary Figures

**Supplementary Figure 1: Odds ratios of COVID-19 hospitalization in the presence versus absence of asthma one study removed sensitivity analysis.**

 **Supplementary Figure 2: Odds ratios of COVID-19 hospitalization in the presence versus absence of COPD one study removed sensitivity analysis.**

**Supplementary Figure 3: Odds ratios of COVID-19 ICU admission in the presence versus absence of asthma one study removed sensitivity analysis.**

**Supplementary Figure 4: Odds ratios of COVID-19 ICU admission in the presence versus absence of COPD one study removed sensitivity analysis.**

**Supplementary Figure 5: Odds ratios of COVID-19 mortality in the presence versus absence of asthma one study removed sensitivity analysis.**

**Supplementary Figure 6: Odds ratios of COVID-19 mortality in the presence versus absence of COPD one study removed sensitivity analysis.**

**Supplementary Figure 7: Hazard ratios of COVID-19 mortality (as determined by Cox regression) in the presence versus absence of asthma one study removed sensitivity analysis.**

**Supplementary Figure 8: Hazard ratios of COVID-19 mortality (as determined by Cox regression) in the presence versus absence of COPD one study removed sensitivity analysis.**

**Supplementary Figure 9: Odds ratios of COVID-19 hospitalization in the presence versus absence of asthma sub-group analysis.**

 **Supplementary Figure 10: Odds ratios of COVID-19 hospitalization in the presence versus absence of COPD sub-group analysis.**

**Supplementary Figure 11: Odds ratios of COVID-19 ICU admission in the presence versus absence of asthma sub-group analysis.**

**Supplementary Figure 12: Odds ratios of COVID-19 ICU admission in the presence versus absence of COPD sub-group analysis.**

**Supplementary Figure 13: Odds ratios of COVID-19 mortality in the presence versus absence of asthma sub-group analysis.**

 **Supplementary Figure 14: Odds ratios of COVID-19 mortality in the presence versus absence of COPD sub-group analysis.**

**Supplementary Figure 15: Hazard ratios of COVID-19 mortality (as determined by Cox regression) in the presence versus absence of asthma sub-group analysis.**

**Supplementary Figure 16: Hazard ratios of COVID-19 mortality (as determined by Cox regression) in the presence versus absence of COPD sub-group analysis.**
