# Supplementary material for: The Risk of COVID-19 Related Hospitalsation, Intensive Care Unit Admission and Mortality in People With Underlying Asthma or COPD: A Systematic Review and Meta-Analysis
Source: Front Med (Lausanne). 2021 Jun 16;8:668808. doi: 10.3389/fmed.2021.668808 (PMC8242585; doi:10.3389/fmed.2021.668808)
Supplement: Supplementary file 2 [file Data_Sheet_2.docx]

**Supplementary Table 1: PRISMA Checklist.**

| **Section and Topic** | **Item #** | **Checklist item** | **Location where item is reported** |
| --- | --- | --- | --- |
| **TITLE** | | |  |
| Title | 1 | Identify the report as a systematic review. | Page 1 |
| **ABSTRACT** | | |  |
| Abstract | 2 | See the PRISMA 2020 for Abstracts checklist. | Page 2 |
| **INTRODUCTION** | | |  |
| Rationale | 3 | Describe the rationale for the review in the context of existing knowledge. | Page 3 |
| Objectives | 4 | Provide an explicit statement of the objective(s) or question(s) the review addresses. | Page 4 |
| **METHODS** | | |  |
| Eligibility criteria | 5 | Specify the inclusion and exclusion criteria for the review and how studies were grouped for the syntheses. | Page 6 |
| Information sources | 6 | Specify all databases, registers, websites, organisations, reference lists and other sources searched or consulted to identify studies. Specify the date when each source was last searched or consulted. | Page 5 |
| Search strategy | 7 | Present the full search strategies for all databases, registers and websites, including any filters and limits used. | Page 5 |
| Selection process | 8 | Specify the methods used to decide whether a study met the inclusion criteria of the review, including how many reviewers screened each record and each report retrieved, whether they worked independently, and if applicable, details of automation tools used in the process. | Page 6 |
| Data collection process | 9 | Specify the methods used to collect data from reports, including how many reviewers collected data from each report, whether they worked independently, any processes for obtaining or confirming data from study investigators, and if applicable, details of automation tools used in the process. | Page 7 |
| Data items | 10a | List and define all outcomes for which data were sought. Specify whether all results that were compatible with each outcome domain in each study were sought (e.g. for all measures, time points, analyses), and if not, the methods used to decide which results to collect. | Page 7 |
|  | 10b | List and define all other variables for which data were sought (e.g. participant and intervention characteristics, funding sources). Describe any assumptions made about any missing or unclear information. | Page 7 |
| Study risk of bias assessment | 11 | Specify the methods used to assess risk of bias in the included studies, including details of the tool(s) used, how many reviewers assessed each study and whether they worked independently, and if applicable, details of automation tools used in the process. | Page 7 |
| Effect measures | 12 | Specify for each outcome the effect measure(s) (e.g. risk ratio, mean difference) used in the synthesis or presentation of results. | Page 7 |
| Synthesis methods | 13a | Describe the processes used to decide which studies were eligible for each synthesis (e.g. tabulating the study intervention characteristics and comparing against the planned groups for each synthesis (item #5)). | Page 7 |
|  | 13b | Describe any methods required to prepare the data for presentation or synthesis, such as handling of missing summary statistics, or data conversions. | Page 7 |
|  | 13c | Describe any methods used to tabulate or visually display results of individual studies and syntheses. | Page 7 |
|  | 13d | Describe any methods used to synthesize results and provide a rationale for the choice(s). If meta-analysis was performed, describe the model(s), method(s) to identify the presence and extent of statistical heterogeneity, and software package(s) used. | Page 7 |
|  | 13e | Describe any methods used to explore possible causes of heterogeneity among study results (e.g. subgroup analysis, meta-regression). | Page 7 |
|  | 13f | Describe any sensitivity analyses conducted to assess robustness of the synthesized results. | Page 8 |
| Reporting bias assessment | 14 | Describe any methods used to assess risk of bias due to missing results in a synthesis (arising from reporting biases). | NA (there were no missing results) |
| Certainty assessment | 15 | Describe any methods used to assess certainty (or confidence) in the body of evidence for an outcome. | Page 7 |
| **RESULTS** | | |  |
| Study selection | 16a | Describe the results of the search and selection process, from the number of records identified in the search to the number of studies included in the review, ideally using a flow diagram. | Page 9 |
|  | 16b | Cite studies that might appear to meet the inclusion criteria, but which were excluded, and explain why they were excluded. | Page 9 |
| Study characteristics | 17 | Cite each included study and present its characteristics. | Pages 10-19 |
| Risk of bias in studies | 18 | Present assessments of risk of bias for each included study. | Page 9 and Supplementary Table 4. |
| Results of individual studies | 19 | For all outcomes, present, for each study: (a) summary statistics for each group (where appropriate) and (b) an effect estimate and its precision (e.g. confidence/credible interval), ideally using structured tables or plots. | Figures 2-5 and Table 2. |
| Results of syntheses | 20a | For each synthesis, briefly summarise the characteristics and risk of bias among contributing studies. | Table 2 |
|  | 20b | Present results of all statistical syntheses conducted. If meta-analysis was done, present for each the summary estimate and its precision (e.g. confidence/credible interval) and measures of statistical heterogeneity. If comparing groups, describe the direction of the effect. | Table 2 and Figures 2-5. |
|  | 20c | Present results of all investigations of possible causes of heterogeneity among study results. | Table 3 and also discussed on page 25. |
|  | 20d | Present results of all sensitivity analyses conducted to assess the robustness of the synthesized results. | Page 20-21 and Supplementary Figures 1-8. |
| Reporting biases | 21 | Present assessments of risk of bias due to missing results (arising from reporting biases) for each synthesis assessed. | Table 2. Page 22 |
| Certainty of evidence | 22 | Present assessments of certainty (or confidence) in the body of evidence for each outcome assessed. | Table 2. Page 22 |
| **DISCUSSION** | | |  |
| Discussion | 23a | Provide a general interpretation of the results in the context of other evidence. | Page 24-25. |
|  | 23b | Discuss any limitations of the evidence included in the review. | Page 25. |
|  | 23c | Discuss any limitations of the review processes used. | Page 25. |
|  | 23d | Discuss implications of the results for practice, policy, and future research. | Page 25. |
| **OTHER INFORMATION** | | |  |
| Registration and protocol | 24a | Provide registration information for the review, including register name and registration number, or state that the review was not registered. | Page 26. |
|  | 24b | Indicate where the review protocol can be accessed, or state that a protocol was not prepared. | Page 26. |
|  | 24c | Describe and explain any amendments to information provided at registration or in the protocol. | Supplementary Table 1. |
| Support | 25 | Describe sources of financial or non-financial support for the review, and the role of the funders or sponsors in the review. | Page 26. |
| Competing interests | 26 | Declare any competing interests of review authors. | Page 26. |
| Availability of data, code and other materials | 27 | Report which of the following are publicly available and where they can be found: template data collection forms; data extracted from included studies; data used for all analyses; analytic code; any other materials used in the review. | Page 26. |

**Supplementary Table 2: Justifications of deviations from the pre-registered protocol.**

| **Section of the protocol that has changed** | **Description of deviation** | **Justification for deviation** |
| --- | --- | --- |
| Review question:  What does the evidence tell us about the risk of infection, the risk of serious illness, and the risk of death for people with COPD and/or asthma infected with SARS-CoV-2? | Question changed to:  What does the evidence tell us about the risk of hospitalization, risk of serious illness, and the risk of death for people with COPD and/or asthma infected with SARS-CoV-2? | After a full-team discussion, it was decided that the term ‘risk of infection’ was too broad and decided to clarify in greater detail. |
| Secondary questions:  1. How does that compare to other systemic conditions?  2. What numbers have been treated in intensive care?  3. What proportion of all COVID-19-related deaths involved COPD and/or asthma as a pre-existing illness? | These secondary questions were abandoned. | After carful consideration, we felt that adding these secondary questions had the potential to make the study too broad, and have instead decided to tackle these other questions in other studies. |
| Measures of effect (whole section) | These were changed to:  We changed this section to only include adjusted effect sizes, whereas the previous version was not specific. | Upon preliminary search, we found several meta-anlyses reporting the risks we were aiming to examine, however none of these reported risks were adjusted for any cofounding variables. It was therefore decided as a team to focus on adjusted effect sizes to give the reader a more robust reporting of risk. |
| Risk of bias assessment | This was changed from the Newcastle-Ottawa Scale to the QUIPS tool. | Upon reflection, we noted that several parts of the NOS would have been irrelevant for the types of study we were looking to include, and the QUIPS was more suitable, therefore was changed. |

**Supplementary Table 3: List of excluded studies with reasons for exclusion**

| Author | Title | Reason for exclusion |
| --- | --- | --- |
| Argenziano et al. | Characterization and clinical course of 1000 patients with coronavirus disease 2019 in New York: retrospective case series. | Dataset unavailable so unable to run analysis |
| Asfaw et al. | PRS65 ADHERENCE AND PERSISTENCE TO ROFLUMILAST AMONG ADULT PATIENTS WITH CHRONIC OBSTRUCTIVE PULMONARY DISEASE | Study not related to COVID-19 |
| Atere et al. | COVID-19: The Case of Three Patients with the Same Diagnosis but Different Clinical and Laboratory Features | Case report |
| Attaway et al. | Management of patients with COPD during the COVID-19 pandemic | No primary data |
| Avdeev et al. | Low prevalence of bronchial asthma and chronic obstructive lung disease among intensive care unit patients with COVID-19 | Dataset unavailable so unable to run analysis |
| Baeza-Martinez et al. | Reduction in Hospital Admissions for COPD Exacerbation During the Covid-19 Pandemic | No primary data |
| Bandi et al. | African American children are at higher risk of COVID-19 infection | No other comorbidities measured |
| Baptist et al. | Asthma disparities during the COVID-19 pandemic: a survey of patients and physicians. | Outcome irrelevant |
| Baranov et al. | Statement on management of children with allergic diseases during new coronaviral infection SARS-CoV-2 pandemic (COVID-19 infection) | Not in English |
| Barbu et al. | The Impact of SARS-CoV-2 on the Most Common Comorbidities-A Retrospective Study on 814 COVID-19 Deaths in Romania | No non-death control group |
| Barroso et al. | Presenting prevalence, characteristics and outcome of asthmatic patients with T2 diseases in hospitalized subjects with COVID-19 in Madrid, Spain | Dataset unavailable so unable to run analysis |
| Barsoum | Pediatric Asthma \& Coronavirus (COVID-19)-Clinical Presentation in an Asthmatic Child-Case Report | Case report |
| Bassendine et al. | COVID-19 and comorbidities: A role for dipeptidyl peptidase 4 (DPP4) in disease severity? | No primary data |
| Beaney et al. | Assessment and management of adults with asthma during the covid-19 pandemic | No primary data |
| Beauchamp Bhutani et al. | Key Highlights of the Canadian Thoracic Society's Position Statement on the Optimization of COPD Management During the Coronavirus Disease 2019 Pandemic. | No primary data |
| Beauchamp Bhutani et al. | Addressing therapeutic questions to help Canadian health care professionals optimize COPD management for their patients during the COVID-19 pandemic | No primary data |
| Bellanti et al. | COVID-19, allergic disease and asthma: Extraordinary challenges for the allergist/immunologist | No primary data |
| Bentivegna et al. | Extremely Severe Case of COVID-19 Pneumonia Recovered Despite Bad Prognostic Indicators: a Didactic Report | Case report |
| Berghaus et al. | Disproportionate decline in admissions for exacerbated COPD during the COVID-19 pandemic | Outcome irrelevant |
| Bergquist et al. | Non-hospitalized Adults with COVID-19 Differ Noticeably from Hospitalized Adults in Their Demographic, Clinical, and Social Characteristics | Dataset unavailable so unable to run analysis |
| Beurnier et al. | Characteristics and outcomes of asthmatic patients with COVID-19 pneumonia who require hospitalisation | Dataset unavailable so unable to run analysis |
| Bhalla et al. | Dupilumab, severe asthma airway responses, and SARS-CoV-2 serology. | Case report |
| Bhandari et al. | Clinical Profile of Covid-19 Infected Patients Admitted in a Tertiary Care Hospital in North India | No primary data available (lead author contact details not available) |
| Bhandari et al. | Clinico-Radiological Evaluation and Correlation of CT Chest Images with Progress of Disease in COVID-19 Patients | Dataset unavailable so unable to run analysis |
| Bhatraju et al. | Covid-19 in Critically Ill Patients in the Seattle Region - Case Series. | Dataset unavailable so unable to run analysis |
| Birkmeyer et al. | The Impact Of The COVID-19 Pandemic On Hospital Admissions In The United States. | Outcome irrelevant |
| Boixeda et al. | Is chronic obstructive pulmonary disease a protective factor in SARS-CoV-2 infection? The importance of bronchodilator treatment | Not in English |
| Bolaki et al. | Respiratory diseases in the era of COVID-19: Pearls and pitfalls | No primary data |
| Borobia et al. | A Cohort of Patients with COVID-19 in a Major Teaching Hospital in Europe | Data insufficient to run analysis |
| Bouquet et al. | Microbial burden and viral exacerbations in a longitudinal multicenter COPD cohort | Study not related to COVID-19 |
| Bousquet et al. | ARIA-EAACI statement on asthma and COVID-19 (June 2, 2020) | No primary data |
| Bradding et al. | ACE2, TMPRSS2, and furin gene expression in the airways of people with asthma-implications for COVID-19 | Outcome irrelevant |
| Bradley et al. | Histopathology and ultrastructural findings of fatal COVID-19 infections in Washington State: a case series | Outcome irrelevant |
| Brendish et al. | Clinical characteristics, symptoms and outcomes of 1054 adults presenting to hospital with suspected COVID-19: a comparison of patients with and without SARS-CoV-2 infection. | Dataset unavailable so unable to run analysis |
| Broadhurst et al. | Asthma in COVID-19 Hospitalizations: An Overestimated Risk Factor? | SR |
| Bruzzese et al. | The paradoxical effect of IL-6 and implications for the use of Tocilizumab in Covid-19 patients | No primary data |
| Burn et al. | An international characterisation of patients hospitalised with COVID-19 and a comparison with those previously hospitalised with influenza. | Preprint/not peer reviewed |
| Butler et al. | Prevalence of comorbid asthma in COVID-19 patients | Preprint/not peer reviewed |
| Cote et al. | Early prediction of the risk of severe coronavirus disease 2019: A key step in therapeutic decision making | Impossible to determine any other co-morbidities |
| Cafferkey et al. | Human rhinovirus infection and COPD: role in exacerbations and potential for therapeutic targets | No primary data |
| Cai et al. | Tobacco smoking increases the lung gene expression of ACE2, the Receptor of SARS-CoV-2 | Outcomes not related to COVID-19 |
| Callender et al. | The Impact of Pre-existing Comorbidities and Therapeutic Interventions on COVID-19 | No primary data |
| Caminati et al. | Asthmatic patients in COVID-19 outbreak: Few cases despite many cases | Reply to paper |
| Canevelli et al. | COVID-19 mortality among migrants living in Italy. | Data insufficient to run analysis |
| Capone | Simultaneous circulation of COVID-19 and flu in Italy: Potential combined effects on the risk of death? | No primary data |
| Caratozzolo et al. | The impact of COVID-19 on health status of home-dwelling elderly patients with dementia in East Lombardy, Italy: results from COVIDEM network | Outcome irrelevant |
| Carli et al. | Is asthma protective against COVID-19? | No primary data |
| Carli et al. | Asthma phenotypes, comorbidities, and disease activity in COVID-19: The need of risk stratification. Reply to Morais-Almeida | Reply to paper |
| Carvalho et al. | Asthma and COVID-19: Current evidence | Full text unavailable |
| Cashman | Why the lower reported prevalence of asthma in patients diagnosed with COVID-19 validates repurposing EDTA solutions to prevent and manage treat COVID-19 disease | No primary data |
| Castro-Rodriguez and Forno | Asthma and COVID-19 in children: A systematic review and call for data | Preprint/not peer reviewed |
| Catizone et al. | One-third of young adults susceptible to severe covid-19 | Full text unavailable |
| Cevhertas et al. | Advances and recent developments in asthma in 2020 | Review |
| Chaabouni et al. | Vitamin D supplementation to prevent COVID-19 in patients with COPD: A research perspective | No primary data |
| Chai et al. | Common concerns in managing bronchial asthma during the COVID-19 pandemic | No primary data |
| Chalubinski et al. | The relationship between human coronaviruses, asthma and allergy - an unresolved dilemma | No primary data |
| Chan et al. | Significant reduction in hospital admissions for acute exacerbation of chronic obstructive pulmonary disease in Hong Kong during coronavirus disease 2019 pandemic. | Outcome irrelevant |
| Chandra et al. | Frequency of comorbidities \& their association with intensive care unit admission in hospitalised patients with 2019 novel coronavirus infection in tertiary care centres of three states of India | Full text unavailable |
| Chang et al. | Asthma control, self-management and healthcare access during the COVID-19 epidemic in Beijing | No other comorbidities measured |
| Chang et al. | Rhinovirus Infections in Individuals with Asthma Increase ACE2 Expression and Cytokine Pathways Implicated in COVID-19. | Outcome irrelevant |
| Chao et al. | Clinical Characteristics and Outcomes of Hospitalized and Critically Ill Children and Adolescents with Coronavirus Disease 2019 at a Tertiary Care Medical Center in New York City | Dataset unavailable so unable to run analysis |
| Chavasse | Covid-19: Reduced asthma presentations in children | No primary data |
| Chavasse et al. | The Indirect Impact of COVID-19 on Children With Asthma | Outcome irrelevant |
| Chow et al. | Preliminary estimates of the prevalence of selected underlying health conditions among patients with coronavirus disease 2019 - United States, February 12-March 28, 2020 | Dataset unavailable so unable to run analysis |
| Ciprandi et al. | Children and adolescents with allergy and/or asthma seem to be protected from coronavirus disease 2019. | No primary data |
| Codispoti et al. | Clinical course of asthma in 4 cases of coronavirus disease 2019 infection. | Case report |
| Collinsworth et al. | PRS52 LEVERAGING EHR DATA TO IDENTIFY CARE IMPROVEMENT OPPORTUNITIES FOR PATIENTS WITH ASTHMA AND COPD IN A LARGE, INTEGRATED HEALTH CARE DELIVERY ORGANIZATION | Outcomes not related to COVID-19 |
| Cote et al. | Early prediction of the risk of severe coronavirus disease 2019: A key step in therapeutic decision making | No primary data |
| Creel-Bulos et al. | Acute cor pulmonale in critically ill patients with covid-19 | Case report |
| Halpin et al. | Do chronic respiratory diseases or their treatment affect the risk of SARS-CoV-2 infection? | No primary data |
| D'Anna et al. | Evaluation of Innate Immune Mediators Related to Respiratory Viruses in the Lung of Stable COPD Patients. | Outcomes not related to COVID-19 |
| Daccord and Touilloux | Asthma and COPD management during the COVID-19 pandemic | Not in English |
| Davis | Using Predictive Scoring Systems for Asthma Exacerbations Could Help Safely Conserve Resources During the COVID-19 Pandemic...Miller AG, Haynes KE, Gates RM, et al. Initial Modified Pulmonary Index Score Predicts Hospital Length of Stay for Asthma Subject | No primary data |
| DeBiasi et al. | Severe Coronavirus Disease-2019 in Children and Young Adults in the Washington, DC, Metropolitan Region | No primary data |
| Deshpande et al. | Study of COVID-19 Pandemic in Representative Dialysis Population Across Mumbai, India: An Observational Multicentric Analysis | Outcomes not relevant and author correspondence details unavailable |
| Deslee et al. | Chronic obstructive pulmonary disease and the COVID-19 pandemic: Reciprocal challenges | No primary data |
| Dominguez-Ortega et al. | Early experiences of SARS-CoV-2 infection in severe asthmatics receiving biologic therapy | Case report |
| Du et al. | Clinical characteristics of 182 pediatric COVID-19 patients with different severities and allergic status. | No asthma and/or COPD measured |
| Duanmu et al. | Characteristics of Emergency Department Patients With COVID-19 at a Single Site in Northern California: Clinical Observations and Public Health Implications. | Dataset unavailable so unable to run analysis |
| Eastin | Characteristics and Outcomes of 21 Critically Ill Patients with COVID-19 in Washington State | Dataset unavailable so unable to run analysis |
| Elbeddini et al. | Amid COVID-19 pandemic: Challenges with access to care for COPD patients | No primary data |
| Elbeddini et al. | Strategies to conserve salbutamol pressurized metered-dose inhaler stock levels amid COVID-19 drug shortage | No primary data |
| Escobar et al. | CLINICOEPIDEMIOLOGICAL CHARACTERISTICS of PATIENTS WHO DIED from COVID-19 at A NATIONAL HOSPITAL of LIMA, Peru | Outcome irrelevant |
| Figueira Gonccalves et al. | Clinical challenges in chronic obstructive pulmonary disease in patients who suffered SARS-CoV-2 infection | No primary data |
| Figueira Gonccalves et al. | Chronic Obstructive Pulmonary Disease and SARS-CoV-2 Infection. What Do We Know so Far? | Not in English |
| Flick et al. | R.; Lamprecht | No primary data |
| Guler | A. A.; {\"{O}}zt{\"{u}}rk | FT unavailable |
| Garcia-Moguel et al. | COVID-19, severe asthma, and biologics | Case report |
| Garcia-Pachon et al. | Asthma and COPD in Hospitalized COVID-19 Patients | Outcome irrelevant |
| Garcia-Pachon et al. | Asthma prevalence in patients with SARS-CoV-2 infection detected by RT-PCR not requiring hospitalization | Outcome irrelevant |
| Garg et al | Hospitazation rates and characteristics of patients hospitalized with laboratory-confirmed coronavirus disease 2019 - NOVID-NET, 14 states, March 1-30, 2020 | Outcome irrelevant |
| Gemes, et al. | M.; Modig | FT unavailable |
| Gibson et al. | Risk for Severe COVID-19 Illness Among Health Care Workers Who Work Directly with Patients | No asthma and/or COPD measured |
| Goyal et al | Clinical characteristics of COVID-19 in New York City. | Outcome irrelevant |
| Grasselli et al | Baseline characteristics and outcomes of 1591 patients infected with SARS-CoV-2 admitted to ICUs of the Lombardy region Italy | Data used in another included paper |
| Guan et al. | Comorbidity and its impact on 1590 patients with COVID-19 in China: a nationwide analysis. | Data insufficient - outcomes are 'composite end-points rather than ICU admission hospitalisation or mortality |
| Guimaraes | COVID-19: Once upon a time in Portugal: A brief atlas of ongoing pandemic Portuguese research | No primary data |
| Guler et al. | COVID-19 in chronic diseases | Outcome irrelevant |
| Gupta et al. | Asthma in children during the COVID-19 pandemic: lessons from lockdown and future directions for management | No primary data |
| Hailay et al. | The burden, admission, and outcomes of COVID-19 among asthmatic patients in Africa: protocol for a systematic review and meta-analysis. | SR |
| Haroun-Diaz et al. | SEVERE ASTHMA DURING THE COVID-19 PANDEMIC: CLINICAL OBSERVATIONS | Case report |
| Hartmann-Boyce et al. | Asthma and COVID-19: review of evidence on risks and management considerations | No primary data |
| Hasan et al. | Use of corticosteroids in asthma and COPD patients with or without COVID-19. | No primary data |
| Hashim et al. | Population Risk Factors for COVID-19 Mortality in 93 Countries | Outcome irrelevant |
| Haydar et al. | Palliative Care Utilization Among Patients With COVID-19 in an Underserved Population: A Single-Center Retrospective Study | Dataset unavailable so unable to run analysis |
| Heffler et al. | COVID-19 in Severe Asthma Network in Italy (SANI) patients: Clinical features, impact of comorbidities and treatments. | Outcome irrelevant |
| Hegde | Does asthma make COVID-19 worse? | No primary data |
| Hepkaya et al. | General Health Status of Asthmatic Children During COVID-19 Pandemic | Outcome irrelevant |
| Higham et al. | Increased ACE2 Expression in Bronchial Epithelium of COPD Patients who are Overweight | Outcome irrelevant |
| Hirshberg et al. | Care of critically ill pregnant patients with coronavirus disease 2019: a case series | Case report |
| Holland et al. | Home-based or remote exercise testing in chronic respiratory disease, during the COVID-19 pandemic and beyond: A rapid review | No primary data |
| Celebioglu et al. | Asthma and covid-19 | No FT available |
| Sin et al. | COVID-19 in COPD: A growing concern | No primary data |
| Olloquequi et al. | COVID-19 Susceptibility in chronic obstructive pulmonary disease | No primary data |
| Yohannes et al. | COPD patients in a COVID-19 society: depression and anxiety | No primary data |
| Huang et al. | Clinical characteristics of laboratory confirmed positive cases of SARS-CoV-2 infection in Wuhan, China: A retrospective single center analysis | Dataset unavailable so unable to run analysis |
| Hubsch et al. | Gesundheitsverhalten und Bedurfnisse von Menschen mit COPD wahrend der COVID-19-Pandemie | Not in English |
| Hurst et al. | SARS-CoV-2 Infections Among Children in the Biospecimens from Respiratory Virus-Exposed Kids (BRAVE Kids) Study. | Outcome irrelevant |
| Ibrahim et al. | SARS-CoV-2 testing and outcomes in the first 30 days after the first case ofCOVID-19 at an Australian children's hospital | Outcome irrelevant |
| Jackson et al. | Association of respiratory allergy, asthma, and expression of the SARS-CoV-2 receptor ACE2 | Outcome irrelevant |
| Jacobs et al. | Increased expression of ACE2, the SARS-CoV-2 entry receptor, in alveolar and bronchial epithelium of smokers and COPD subjects | No primary data |
| Jain et al. | Predictive symptoms and comorbidities for severe COVID-19 and intensive care unit admission: a systematic review and meta-analysis. | SR |
| Johnston | Asthma and COVID-19: Is asthma a risk factor for severe outcomes? | No primary data |
| Jordan et al. | Covid-19: Risk factors for severe disease and death | No primary data |
| Joshi et al. | Successful treatment of coronavirus disease 2019 in a patient with asthma. | Case report |
| Kabesch | Shielding against SARS-CoV-2 infection is not justified in children with severe asthma. | No primary data |
| Kamali et al. | Challenges in a child with asthma and COVID-19 | Case report |
| Karagiannidis et al. | Case characteristics, resource use, and outcomes of 10 021 patients with COVID-19 admitted to 920 German hospitals: an observational study | Data insufficient and dataset unavailable |
| Kaye et al. | Changes in medication adherence among patients with asthma and COPD during the COVID-19 pandemic | Outcome irrelevant |
| Kenyon et al. | Initial effects of the COVID-19 pandemic on pediatric asthma emergency department utilization | Outcome irrelevant |
| Kim et al. | Management of severe asthma during the COVID-19 pandemic in Korea | No primary data |
| Klimek et al. | Wolfram; Rosenkranz | No primary data |
| Konopka et al. | Postmortem Lung Findings in a Patient With Asthma and Coronavirus Disease 2019. | Case report |
| Kouri et al. | CHEST Reviews: Addressing reduced laboratory-based pulmonary function testing during a pandemic | No primary data |
| Kow et al. | The Potential Benefit of Telmisartan to Protect Overweight Patients with COPD from the Acquisition of COVID-19 | No primary data |
| Kow et al. | Are severe asthma patients at higher risk of developing severe outcomes from COVID-19? | No primary data |
| Krass et al. | COVID-19 Outbreak Among Adolescents at an Inpatient Behavioral Health Hospital. | Outcome irrelevant |
| Krivec et al. | COVID-19 lockdown dropped the rate of paediatric asthma admissions | No primary data |
| Kumar et al. | Management of Asthma in Children during COVID-19 Pandemic | No primary data |
| Kuzeva et al. | Time-critical administration of corticosteroid rescue therapy for COVID-19 pneumonitis in a ward-based patient with chronic obstructive pulmonary disease | Case report |
| Rezende et al. | Adults at high-risk of severe coronavirus disease-2019 (Covid-19) in Brazil | Outcome irrelevant |
| Laires et al. | Population-based Estimates for High Risk of Severe COVID-19 Disease due to Age and Underlying Health Conditions. | Outcome irrelevant |
| Le et al. | PRS11 HEALTHCARE RESOURCE UTILIZATION AND COSTS IN OLDER COPD PATIENTS WITH AND WITHOUT FREQUENT EXACERBATIONS | Outcomes not related to COVID-19 |
| Lee et al. | Epidemiological and clinical characteristics of coronavirus disease 2019 in Daegu, South Korea | Dataset unavailable so unable to run analysis |
| Lemus Calderon et al. | Differentiating characteristics of patients with asthma in the severe acute respiratory syndrome coronavirus 2 infection | Full text unavailable |
| Leung et al. | COVID-19 and COPD | SR |
| Leung et al. | ACE-2 expression in the small airway epithelia of smokers and COPD patients: implications for COVID-19 | No primary data |
| Levin et al. | Acute asthma management during SARS-CoV2-pandemic 2020. | No primary data |
| Li et al. | Risk factors for severity and mortality in adult COVID-19 inpatients in Wuhan. | No asthma and/or COPD measured |
| Lian et al. | High neutrophil-to-lymphocyte ratio associated with progression to critical illness in older patients with COVID-19: a multicenter retrospective study. | Dataset unavailable so unable to run analysis |
| Licari et al. | Allergy and asthma in children and adolescents during the COVID outbreak: What we know and how we could prevent allergy and asthma flares | No asthma and/or COPD measured |
| Licari et al. | Biologic Use in Allergic and Asthmatic Children and Adolescents During the COVID-19 Pandemic. | Outcome irrelevant |
| Licskai et al. | Addressing therapeutic questions to help Canadian physicians optimize asthma management for their patients during the COVID-19 pandemic | No primary data |
| Licskai et al. | Key Highlights From the Canadian Thoracic Society Position Statement on the Optimization of Asthma Management During the Coronavirus Disease 2019 Pandemic. | No primary data |
| Lieberman-Cribbin et al. | The Impact of Asthma on Mortality in Patients With COVID-19 | No other comorbidities measured |
| Lin et al | The mental health effects of COVID-19 on health care providers in China | No primary data |
| Lippi et al. | Chronic obstructive pulmonary disease is associated with severe coronavirus disease 2019 (COVID-19). | SR |
| Lipworth and Chan | Predicting Severe Outcomes in COVID-19 | Narrative review - no primary data |
| Lipworth et al. | Inhaled Corticosteroids and COVID-19 | No primary data |
| Lipworth et al. | Use of inhaled corticosteroids in asthma and coronavirus disease 2019: Keep calm and carry on. | No primary data |
| Liu et al. | Risk factors for developing into critical COVID-19 patients in Wuhan, China: A multicenter, retrospective, cohort study. | Dataset unavailable so unable to run analysis |
| Liu et al. | Clinical outcomes of COVID-19 in Wuhan, China: a large cohort study | Dataset unavailable so unable to run analysis |
| Liu et al. | COVID-19 and Asthma: Reflection During the Pandemic. | No primary data |
| Lombardi et al. | Clinical course and outcomes of patients with asthma hospitalized for severe acute respiratory syndrome coronavirus 2pneumonia: A single-center, retrospective study. | Incomplete dataset |
| Lommatzsch et al. | COVID-19 in a patient with severe asthma treated with Omalizumab | Case report |
| Lopez-Campos et al. | Telephone Support for Copd Patients During COVID-19 | No primary data |
| Lovinsky-Desir et al. | Asthma among hospitalized patients with COVID-19 and related outcomes. | Outcomes irrelevant |
| Ma et al. | Is asthma an exclusionary condition for SARS-CoV-2 infection in China? -the relationship between asthma and COVID-19. | SR |
| Maes et al. | COVID-19, asthma, and inhaled corticosteroids: Another beneficial effect of inhaled corticosteroids? | No primary data |
| Mahdavinia et al. | Does asthma affect outcomes of patients with COVID-19 infections? | No primary data |
| Martinez et al. | Role of infection and antimicrobial therapy in acute exacerbations of chronic obstructive pulmonary disease | No primary data |
| Matsumoto et al. | Does asthma affect morbidity or severity of COVID-19? | Narrative review - no primary data |
| Matucci et al. | COVID-19 in severe asthmatic patients during ongoing treatment with biologicals targeting type 2 inflammation: Results from a multicenter Italian survey | No non-asthma control |
| Matusiak et al. | Expression of SARS-CoV-2 entry receptors in the respiratory tract of healthy individuals, smokers and asthmatics. | Outcome irrelevant |
| Medicine, The Lancet Respiratory | Reflecting on World Asthma Day in the era of COVID-19 | No primary data |
| Mei-Zahav et al. | Aerosol treatments for childhood asthma in the era of COVID-19 | No primary data |
| Mendy et al. | Factors Associated with Hospitalization and Disease Severity in a Racially and Ethnically Diverse Population of COVID-19 Patients. | Preprint/not peer reviewed |
| Meyer | Transmission, start of symptom and morbidity among Danish COVID-19 patient s admitted to hospital | Not enough data to run analysis |
| Miller et al. | Clinical presentation and course of COVID-19 | No primary data |
| Miwa et al. | Peripheral Swiss Cheese" appearance in a COVID-19 patient with chronic obstructive pulmonary disease" | Case report |
| Mo et al. | Abnormal pulmonary function in COVID-19 patients at time of hospital discharge | Outcome irrelevant |
| Morais-Almeida et al. | Update on asthma prevalence in severe COVID-19 patients | No primary data |
| Morais-Almeida et al. | COVID-19 and asthma: To have or not to have T2 inflammation makes a difference? | No primary data |
| Morais-Almeida et al. | COVID-19, asthma, and biological therapies: What we need to know. | No primary data |
| Murphy et al. | Effects of Asthma and Human Rhinovirus A16 on the Expression of the SARS-CoV-2 Entry Factors in Human Airway Epithelium | Outcome irrelevant |
| Papadoppulos et al. | Impact of COVID-19 on Pediatric Asthma: Practice Adjustments and Disease Burden. | Outcome irrelevant |
| Nagakumar et al. | Acute asthma management considerations in children and adolescents during the COVID-19 pandemic | No primary data |
| Niquini et al. | Description and comparison of demographic characteristics and comorbidities in SARI from COVID-19, SARI from influenza, and the Brazilian general population. | Dataset unavailable so unable to run analysis |
| Novak et al. | Viruses and asthma: the role of common respiratory viruses in asthma and its potential meaning for SARS-CoV-2 | No primary data |
| Nystad et al. | Underlying conditions in adults with COVID-19. | Dataset unavailable so unable to run analysis |
| Olaguibel et al. | Upper and Lower Airways Functional Examination in Asthma and Respiratory Allergic Deseases. Considerations in the SARS-CoV-2 Post-Pandemic Situation | No primary data |
| Ono et al. | Asthma exacerbation associated with COVID-19 pneumonia | Case report |
| Oreskovic et al. | The Unexpected Risks of COVID-19 on Asthma Control in Children | Outcome irrelevant |
| Otto et al. | The Epidemiology of SARS-CoV-2 in a Pediatric Healthcare Network in the United States | Outcome irrelevant |
| Ozturk et al. | A. B.; {\c{C}}aƒülayan | FT unavailable |
| Palmieri et al. | Clinical Characteristics of Hospitalized Individuals Dying With COVID-19 by Age Group in Italy | Outcome irrelevant |
| Papadopoulos, et al. | Mika J.; Morais-Almeida | FT unavailable |
| Patrucco et al. | Severe asthma at COVID-19 time: what's new on biologic therapies | No primary data |
| Pedrozo-Pupo et al. | Depression, perceived stress related to COVID, post-traumatic stress, and insomnia among asthma and COPD patients during the COVID-19 pandemic. | Outcome irrelevant |
| Pennington | Asthma increases risk of severity of COVID-19 | No primary data |
| Perincek and Avci | A Case Series of Patients with COVID-19 Infection Admitted to a Secondary Care Center in Turkey | Data insufficient to run analysis |
| Peters et al. | COVID-19-related genes in sputum cells in asthma: Relationship to demographic features and corticosteroids | Outcome irrelevant |
| Philip et al. | Respiratory patient experience of measures to reduce risk of COVID-19: findings from a descriptive cross-sectional UK wide survey. | Outcome irrelevant |
| Pinto et al. | ACE2 expression is increased in the lungs of patients with comorbidities associated with severe COVID-19 | Outcome irrelevant |
| Poblador-Plou et al. | Baseline chronic comorbidity and mortality in laboratory-confirmed COVID-19 cases: Results from the PRECOVID study in Spain | Dataset unavailable so unable to run analysis |
| Poggiali et al. | COVID-19, chronic obstructive pulmonary disease and pneumothorax: A frightening triad | Case report |
| Pranata et al. | Effect of chronic obstructive pulmonary disease and smoking on the outcome of COVID-19 | SR |
| Press et al. | Concerns About Coronavirus Disease-Related Collateral Damage for Patients With COPD | No primary data |
| Renner et al. | COVID-19 in a severe eosinophilic asthmatic receiving benralizumab-a case study | Case report |
| Richardson et al | Presenting characteristics, comorbidities, and outcomes among 5700 patients hospitalised with COVID-19 in the New York City area. | Dataset unavailable so unable to run analysis |
| Rogala et al. | Biological therapy of asthma - position statement of Polish allergology society (Pta) and polish society of lung diseases (ptchp) | Not in English |
| Rong et al. | PRS57 ASSOCIATION BETWEEN TRANSIENT OPIOID USE AND ACUTE COPD EXACERBATION AMONG OLDER ADULTS WITH CHRONIC OBSTRUCTIVE PULMONARY DISEASE (COPD) | Conference abstract |
| Rossato et al. | Current smoking is not associated with COVID-19 | No primary data |
| Ruano et al. | Impact of the COVID-19 pandemic in children with allergic asthma | No non-asthma control |
| Russell | Covid-19 and COPD: A personal reflection | No primary data |
| Saheb Sharif-Askari et al. | Airways Expression of SARS-CoV-2 Receptor, ACE2, and TMPRSS2 Is Lower in Children Than Adults and Increases with Smoking and COPD | Outcome irrelevant |
| Sahu et al. | COVID-19 and restrictive lung disease: A deadly combo to trip off the fine balance | Case report |
| Salacup et al. | Characteristics and clinical outcomes of COVID-19 patients in an underserved-inner city population: A single tertiary center cohort | Dataset unavailable so unable to run analysis |
| Sanchez-Ramirez et al. | Underlying respiratory diseases, specifically COPD, and smoking are associated with severe COVID-19 outcomes: A systematic review and meta-analysis. | SR |
| Sandalci and Uyaroglu | The role and importance of chronic diseases in COVID-19 and related recommendations | Not in English |
| Sarƒoƒülu and Sarioglu | Asthma and COVID-19: What do we know? | Narrative review - no primary data |
| Satia et al. | Prevalence and contribution of respiratory viruses in the community to rates of emergency department visits and hospitalizations with respiratory tract infections, chronic obstructive pulmonary disease and asthma | Outcomes not related to COVID-19 |
| Schultze et al. | Risk of COVID-19-related death among patients with chronic obstructive pulmonary disease or asthma prescribed inhaled corticosteroids: an observational cohort study using the OpenSAFELY platform. | No non-COPD control group |
| See et al. | COVID-19: Four Paediatric Cases in Malaysia | Case report |
| Shi et al. | Prediction of adverse clinical outcomes in patients with coronavirus disease 2019 | No asthma and/or COPD measured |
| Shi et al. | Clinical characteristics of COVID-19 patients combined with allergy | No asthma and/or COPD measured |
| Shukla et al. | COVID-19 and chronic obstructive pulmonary disease: therapeutic potential of blocking SARS-CoV2 adhesion factors | No primary data |
| Silver et al. | Clinical Characteristics and Outcomes Based on Race of Hospitalized Patients With COVID-19 in a New Orleans Cohort. | Outcome irrelevant |
| Simons et al. | Caring for patients with COPD and COVID-19: A viewpoint to spark discussion | No primary data |
| Skevaki et al. | Asthma-associated risk for COVID-19 development. | No primary data |
| Smith-Ray et al. | Distribution of Patients at Risk for Complications Related to COVID-19 in the United States: Model Development Study. | No asthma and/or COPD measured |
| Somani et al. | Characterization of Patients Who Return to Hospital Following Discharge from Hospitalization for COVID-19 | Outcomes irrelevant |
| Song et al. | Distinct effects of asthma and COPD comorbidity on disease expression and outcome in patients with COVID-19. | Dataset unavailable so unable to run analysis |
| Spinner and Schuldt | A moderate case of COVID-19 viral pneumonia during the sars-CoV-2 pandemic | Case report |
| Ssentongo et al. | Association of cardiovascular disease and 10 other pre-existing comorbidities with COVID-19 mortality: A systematic review and meta-analysis. | SR |
| Stolz et al. | Time-course of upper respiratory tract viral infection and COPD exacerbation | No non-COPD control group |
| Stone, et al. | Case 23-2020: A 76-year-old woman who died from COVID-19 | Case report |
| Strauss | Subcutaneous terbutaline as an alternative to metered-dose inhalers for asthma during the COVID-19 pandemic. | No primary data |
| Sun et al. | Characteristics and prognostic factors of disease severity in patients with COVID-19: The Beijing experience | Dataset unavailable so unable to run analysis |
| Taille et al. | Management of asthma during the Coronavirus disease 2019 outbreak | No primary data |
| Tal-Singer et al. | COPD at the time of COVID-19: A COPD foundation perspective | No primary data |
| Tal-Singer et al. | The COPD Foundation Coronavirus Disease 2019 International Medical Experts Survey: Results. | No primary data |
| Taquechel et al. | Pediatric Asthma Health Care Utilization, Viral Testing, and Air Pollution Changes During the COVID-19 Pandemic. | Outcome irrelevant |
| Taramarcaz et al. | Chronic obstructive pulmonary disease and infection [Broncho-pneumopathie chronique obstructive et infection] | Not in English |
| Temkin-Greener et al. | COVID-19 Pandemic in Assisted Living Communities: Results from Seven States. | Outcomes irrelevant |
| Tran et al. | COVID-19-related perceptions, context and attitudes of adults with chronic conditions: Results from a cross-sectional survey nested in the ComPaRe e-cohort. | Outcomes irrelevant |
| Underner et al. | Asthma and COVID-19: a risk population? | Not in English |
| Upadhya et al. | Hospital treatment of severe acute exacerbation of chronic obstructive pulmonary disease in COVID-19 situation: back to basics | No primary data |
| Uzzaman et al. | Continuing professional education for general practitioners on chronic obstructive pulmonary disease: feasibility of a blended learning approach in Bangladesh. | Outcomes irrelevant |
| van den Bemt and Chavannes | A patient with COPD during the COVID-19 pandemic | Case report |
| Vazquez-Nava et al. | Risk factors of non-adherence to guidelines for the prevention of COVID-19 among young adults with asthma in a region with a high risk of a COVID-19 outbreak | Outcomes irrelevant |
| Verduyn et al. | Serum IgG Concentrations in Adult Patients Experiencing Virus-Induced Severe Asthma Exacerbations | Outcomes irrelevant |
| Violi et al. | IS ALBUMIN PREDICTOR OF MORTALITY IN COVID-19 ? | Dataset unavailable so unable to run analysis |
| Vultaggio et al. | Prompt Predicting of Early Clinical Deterioration of Moderate-to-Severe COVID-19 Patients: Usefulness of a Combined Score Using IL-6 in a Preliminary Study | No asthma and/or COPD measured |
| Guan et al. | Comorbidity and its impact on 1,590 patients with Covid-19 in China: A nationwide analysis | Data insufficient to run analysis |
| Wall-Haas | Connect, Engage: Televists for Children With Asthma During COVID-19. | No primary data |
| Wang, Jian et al. | Clinical and CT findings of COVID-19: differences among three age groups. | Outcomes irrelevant |
| Wang et al.: | COVID-19 and Asthma, the Good or the Bad? | No primary data |
| Wang et al. | Risk factors for hospitalization, intensive care, and mortality among patients with asthma and COVID-19 | No non-asthma control |
| Wang et al. | Treating asthma in the COVID-19 pandemic | No primary data |
| Wang et al. | Does Asthma Increase the Mortality of Patients with COVID-19?: A Systematic Review and Meta-Analysis | SR |
| Wang et al. | The association between COVID-19 and asthma: A systematic review and meta-analysis | SR |
| Wiemers et al. | Disparities in vulnerability to complications from COVID-19 arising from disparities in preexisting conditions in the United States | Outcomes irrelevant |
| Wu, et al. | Risk Factors Associated With Long-Term Hospitalization in Patients With COVID-19: A Single-Centered, Retrospective Study | No asthma and/or COPD measured |
| Lei et al. | Reports Outline COVID-19 Study Findings from Army Medical University ( Risk factors for disease progression in patients with mild to moderate coronavirus disease 2019-a multi- centre observational study ). | No FT available |
| Li et al. | Clinical and Transmission Characteristics of Covid-19 - A Retrospective Study of 25 Cases from a Single Thoracic Surgery Department. | Data insufficient to run analysis |
| Yang et al. | Allergic disorders and susceptibility to and severity of COVID-19: A nationwide cohort study | Outcomes not stratified according to our outcomes |
| Yasudo et al. | Seasonal alteration of isolated pathogens in children hospitalized with asthma exacerbation | Conference abstract |
| Yoo et al. | The role of respiratory viral infections in exacerbation of asthma and chronic obstructive pulmonary disease (COPD) | Not in English |
| Yormaz et al. | Differences of viral panel positive versus negative by real-time PCR in COPD exacerbated patients | Outcomes irrelevant |
| Yu et al. | Patients with COVID-19 in 19 ICUs in Wuhan, China: A cross-sectional study | Outcomes irrelevant |
| Zhang et al. | Cezmi A.; Gao | No asthma and/or COPD measured |
| Zhao et al. | The impact of COPD and smoking history on the severity of COVID-19: A systemic review and meta-analysis | SR |
| Swann et al. | Clinical characteristics of children and young people hospitalised with covid-19 in the United Kingdom: prospective multicentre observational cohort study | No comorbidities measured |
| Long | A picture of severe COVID-19 in US children and youth emerges | No primary data |
| Medicine, The Lancet Respiratory | COVID-19 casts light on respiratory health inequalities | No primary data |
| Krutikov et al. | COVID-19 in care homes (VIVALDI) | No primary data |
| Sha et al. | Expert recommendations on the management of childhood bronchial asthma during the novel coronavirus pneumonia epidemic. | No primary data |
| Stevens | Lessons from covid-19: Visiting patients at home and assessing comorbidities | No primary data |
| Knight et al. | Risk stratification of patients admitted to hospital with covid-19 using the ISARIC WHO Clinical Characterisation Protocol: development and validation of the 4C Mortality Score | No primary data |
| Boyer et al. | COVID-19, medication-assisted treatment, and increased risk for further respiratory depression | Outcomes irrelevant |
| Bin et al. | Environmental Contamination and Viral Shedding in MERS Patients during MERS-CoV Outbreak in South Korea | Outcomes irrelevant |
| Nurmagambetov | PRS49 THE IMPACT OF ASTHMA ON ANNUAL PER-CAPITA ALL-CAUSE HEALTHCARE UTILIZATION AND MEDICAL EXPENDITURE IN CHILDREN | Outcomes irrelevant |
| Millar et al. | Robust, reproducible clinical patterns in hospitalised patients with COVID-19 (preprint) | Preprint/not peer reviewed |
| Nogueira et al | The role of health preconditions on covid-19 deaths in Portugal: Evidence from surveillance data of the first 20293 infection cases | Data inaccurate - 95% CIs were not symmetrical so could not be used in the MA |
| Nanda et a. | A Midwest COVID-19 Cohort for the Evaluation of Multimorbidity and Adverse Outcomes from COVID-19. | Data insufficient |
| Luo et al. | Age differences in clinical features and outcomes in patients with COVID-19, Jiangsu, China: A retrospective, multicentre cohort study | Data insufficient |
| Kow et al. | Are severe asthma patients at higher risk of developing severe outcomes from COVID-19? | Correspondence - no primary data |
| Yan et al. | Association between ambient air pollution and mortality from chronic obstructive pulmonary disease in Wuhan, China: a population-based time-series study. | Data insufficient |
| Zhu et al. | Association of asthma and its genetic predisposition with the risk of severe COVID-19 | Data insufficient |
| Yin et al. | Association of Cigarette Smoking, COPD, and Lung Cancer With Expression of SARS-CoV-2 Entry Genes in Human Airway Epithelial Cells |  |
| Sutter et al. | Association of diabetes and outcomes in patients with COVID-19: Propensity score-matched analyses from a French retrospective cohort | No asthma or COPD data |
| Liao et al. | Association of inhaled and systemic corticosteroid use with Coronavirus Disease 2019 (COVID-19) test positivity in patients with chronic pulmonary diseases | Data insufficient |
| Adrish et al. | Association of smoking status with outcomes in hospitalised patients with COVID-19 | Data insufficient |
| Lovinsky-Desir et al. | Asthma among hospitalized patients with COVID-19 and related outcomes. | Data insufficient |
| Beken et al. | Asthma and allergic diseases are not risk factors for hospitalization in children with coronavirus disease 2019 | Children |
| Garcia-Pachon et al. | Asthma and COPD in hospitalized COVID-19 patients | Data insufficient |
| Ono et al. | Asthma exacerbation associated with COVID-19 pneumonia. | Data insufficient |
| Caminatilli et al. | Asthma in a large COVID-19 cohort: Prevalence, features, and determinants of COVID-19 disease severity. | Data insufficient |
| Carli et al. | Asthma phenotypes, comorbidities, and disease activity in COVID-19: The need of risk stratification. | Correspondence - no primary data |
| Alberca et al. | Asthmatic patients and COVID-19:Â Different disease course? | Data insufficient |
| Best et al. | Baseline Demographics and Clinical Characteristics Among 3471 US Patients Hospitalized with COVID-19 and Pulmonary Involvement: A Retrospective Study | Data insufficient |
| Phelps et al. | Cardiovascular comorbidities as predictors for severe COVID-19 infection or death | Data insufficient |
| Salacup et al. | Characteristics and clinical outcomes of COVID-19 patients in an underserved-inner city population: A single tertiary center cohort. | No data on what adjustments were |
| Goel et al. | Characteristics of COVID-19 at a non-COVID tertiary pulmonary care centre in Delhi, India | Data insufficient |
| Verma et al. | Characteristics of Hospitalized Children With SARS-CoV-2 in the New York City Metropolitan Area. | Data insufficient |
| Zhou et al. | Characteristics of mortal COVID-19 cases compared to the survivors | Review (no primary data) |
| Monday et al. | Characteristics, clinical course, and outcomes of veterans admitted with Covid-19 in Detroit, Michigan | Data insufficient |
| Jimenez et al. | Characteristics, complications and outcomes among 1549 patients hospitalised with COVID-19 in a secondary hospital in Madrid, Spain: A retrospective case series study | Data insufficient |
| Argenziano et al. | Characterization and clinical course of 1000 Patients with COVID-19 in New York: retrospective case series. | Published before Sept 2020 |
| Kim et al. | Characterization of asthma and risk factors for delayed SARS-CoV-2 clearance in adult COVID-19 inpatients in Daegu. | Data insufficient |
| Monterrubio-Flores et al. | Characterizing a two-pronged epidemic in Mexico of non-communicable diseases and SARS-Cov-2: factors associated with increased case-fatality rates. | Insufficient data |
| Martos-Benitez et al. | Chronic comorbidities and clinical outcomes in patients with and without COVID-19: a large population-based study using national administrative healthcare open data of Mexico | Data insufficient - 95% intervals not symmetrical |
| Figueira Gonccalves et al. | Clinical challenges in chronic obstructive pulmonary disease in patients who suffered SARS-CoV-2 infection | Editorial - no primary data |
| Boytsov et al. | Clinical Characteristics and Factors Associated with Poor Outcomes in Hospitalized Patients with Novel Coronavirus Infection COVID-19 | Not in English |
| Cui et al. | Clinical Characteristics and Immune Responses of 137 Deceased Patients With COVID-19: A Retrospective Study | No data on what adjustments were |
| Alnajjar et al. | Clinical characteristics and outcomes of children with COVID-19 in Saudi Arabia. | Review (no primary data) |
| He et al. | Clinical Characteristics and Outcomes of Patients with Severe COVID-19 and Chronic Obstructive Pulmonary Disease (COPD). | Already included in previous analysis |
| Gomez Antunez et al. | Clinical Characteristics and Prognosis of COPD Patients Hospitalized with SARS-CoV-2 | Data insufficient |
| Parra-Bracamonte et al. | Clinical characteristics and risk factors for mortality of patients with COVID-19 in a large data set from Mexico | Already included in previous analysis |
| Xu et al. | Clinical Characteristics and Risk Factors of Cardiac Involvement in COVID-19 | Data insufficient |
| Rial et al. | Clinical characteristics in 545 patients with severe asthma on biological treatment during the COVID-19 outbreak. | Data insufficient |
| Du et al. | Clinical characteristics of 182 pediatric COVID-19 patients with different severities and allergic status. | Data insufficient |
| Riou et al. | Clinical characteristics of and outcomes for patients with COVID-19 and comorbid lung diseases primarily hospitalized in a conventional pulmonology unit: A retrospective study | Data insufficient |
| Yao et al. | Clinical characteristics of COVID-19 patients in three consecutive generations of spread in Zhejiang, China. | Data insufficient |
| Deng et al. | Clinical characteristics of fatal and recovered cases of coronavirus disease 2019 in Wuhan, China: a retrospective study | Data insufficient |
| Chaudhary et al. | Clinical Characteristics, Respiratory Mechanics, and Outcomes in Critically Ill Individuals With COVID-19 Infection in an Underserved Urban Population. | Data insufficient |
| Brendish et al. | Clinical characteristics, symptoms and outcomes of 1054 adults presenting to hospital with suspected COVID-19: a comparison of patients with and without SARS-CoV-2 infection. | Data insufficient |
| Feng et al. | Clinical factors associated with progression and prolonged viral shedding in COVID-19 patients: A multicenter study | Data insufficient |
| Kolin et al. | Clinical, regional, and genetic characteristics of Covid-19 patients from UK Biobank | Data insufficient |
| Thiabaud et al. | Cohort profile: SARS-CoV-2/COVID-19 hospitalised patients in Switzerland. | Data insufficient |
| Badawi et al. | Comparative profile for COVID-19 cases from China and North America: Clinical symptoms, comorbidities and disease biomarkers | Review (no primary data) |
| Miro et al. | Comparison of the demographic characteristics and comorbidities of patients with COVID-19 who died in Spanish hospitals based on whether they were or were not admitted to an intensive care unit. | Data insufficient |
| Shevel | Conditions favoring increased COVID-19 morbidity and mortality: Their common denominator and treatment | Editorial - no primary data |
| Gerayeli et al. | COPD and the risk of poor outcomes in COVID-19: A systematic review and meta-analysis. | Review (no primary data) |
| McAuley et al. | COPD in the time of COVID-19: an analysis of acute exacerbations and reported behavioural changes in patients with COPD. | Data insufficient |
| Pezzuto et al. | COPD influences survival in patients affected by COVID-19, comparison between subjects admitted to an internal medicine unit, and subjects admitted to an intensive care unit: An Italian experience. | Data insufficient |
| Ejaz et al. | COVID-19 and comorbidities: Deleterious impact on infected patients | Data insufficient |
| Clawson et al. | COVID-19 Impacts on Families of Color and Families of Children With Asthma. | Data insufficient |
| Heffler et al. | COVID-19 in Severe Asthma Network in Italy (SANI) patients: Clinical features, impact of comorbidities and treatments. | Data insufficient |
| Matucci et al. | COVID-19 in severe asthmatic patients during ongoing treatment with biologicals targeting type 2 inflammation: Results from a multicenter Italian survey. | Data insufficient |
| Green et al. | COVID-19 Susceptibility in Bronchial Asthma. | Data insufficient |
| Wiertz et al. | COVID-19: patient characteristics in the first phase of post-intensive care rehabilitation. | Data insufficient |
| Junior et al. | Death trends based on autopsy data compared to the beginning of the coronavirus pandemic in Brazil | Data insufficient |
| Lemus Calderon, et al. | Differentiating characteristics of patients with asthma in the severe acute respiratory syndrome coronavirus 2 infection. | Data insufficient |
| Song et al. | Distinct effects of asthma and COPD comorbidity on disease expression and outcome in patients with COVID-19 | Data insufficient |
| Choi et al. | Effect of asthma and asthma medication on the prognosis of patients with COVID-19. | Data insufficient |
| Mash et al. | Evaluation of patient characteristics, management and outcomes for COVID-19 at district hospitals in the Western Cape, South Africa: Descriptive observational study | No asthma or COPD data |
| Fan et al. | Frequency of asthma exacerbation in children during the coronavirus disease pandemic with strict mitigative countermeasures. | Data insufficient |
| Novak et al. | Health Conditions Associated With Severe Illness From COVID-19 Among Individuals With Serious Mental Illness. | Data insufficient |
| Oh et al. | Impact of coronavirus disease-2019 on chronic respiratory disease in South Korea: an NHIS COVID-19 database cohort study | No data on what adjustments were |
| Koff et al. | Impact of Proactive Integrated Care on Chronic Obstructive Pulmonary Disease. | Data insufficient |
| Hernandez-Galdamez, et al. | Increased Risk of Hospitalization and Death in Patients with COVID-19 and Pre-existing Noncommunicable Diseases and Modifiable Risk Factors in Mexico. | Already included in previous analysis |
| Boixeda et al.. | Is chronic obstructive pulmonary disease a protective factor in SARS-CoV-2 infection? The importance of bronchodilator treatment | Data insufficient |
| Avdeev et al. | Low prevalence of bronchial asthma and chronic obstructive lung disease among intensive care unit patients with COVID-19 | Data insufficient |
| Murillo-Zamora, et al. | Predictors of severe symptomatic laboratory-confirmed SARS-CoV-2 reinfection. | Data insufficient |
| Atkins et al. | Preexisting Comorbidities Predicting COVID-19 and Mortality in the UK Biobank Community Cohort. | Already included in previous analysis |
| Signes-Costa et al. | Prevalence and 30-Day Mortality in Hospitalized Patients With Covid-19 and Prior Lung Diseases. | Data insufficient |
| Jungo et al. | Prevalence and risk indicators of first-wave COVID-19 among oral health-care workers: A French epidemiological survey. | Data insufficient |
| Boari, et al. | Prognostic factors and predictors of outcome in patients with COVID-19 and related pneumonia: a retrospective cohort study | ES insufficient |
| Maestre-Muniz et al. | Prognostic Factors at Admission for In-Hospital Mortality from COVID-19 Infection in an Older Rural Population in Central Spain. | Insufficient data |
| Islam et al. | Risk factors associated with morbidity and mortality outcomes of COVID-19 patients on the 28th day of the disease course: A retrospective cohort study in Bangladesh | Data insufficient - no 95% Cis given and not clear if RRs are adjusted |
| Grasselli et al. | Risk Factors Associated With Mortality Among Patients With COVID-19 in Intensive Care Units in Lombardy, Italy. | Already included in previous analysis |
| Tutiya et al. | Risk factors for severe and critical Covid-19 in pregnant women in a single center in Brazil. | Data insufficient |
| Graff et al. | Risk Factors for Severe COVID-19 in Children. | Children |
| Kim et al. | Risk factors on the progression to clinical outcomes of covid-19 patients in south korea: Using national data | Data insufficient |
| Alizadehsani et al. | Risk factors prediction, clinical outcomes, and mortality inÂ COVID-19 patients. | Data insufficient |
| Schultze et al. | Risk of COVID-19-related death among patients with chronic obstructive pulmonary disease or asthma prescribed inhaled corticosteroids: an observational cohort study using the OpenSAFELY platform. | Data insufficient |
| Antonicelli et al. | Severe asthma in adults does not significantly affect the outcome of COVID-19 disease: Results from the Italian Severe Asthma Registry. | Data insufficient |
| Hansen et al. | Severe outcomes of COVID-19 among patients with COPD and asthma. | Data insufficient |
| Caliskan, et al. | Smoking and comorbidities are associated with COVID-19 severity and mortality in 565 patients treated in Turkey: A retrospective observational study | No data on what adjustments were |
| Knight et al. | Symptoms and Clinical Outcomes of Coronavirus Disease 2019 in the Outpatient Setting. | Data insufficient |
| Dong et al. | The clinical characteristics and prognosis of COVID-19 patients with comorbidities: a retrospective analysis of the infection peak in Wuhan. | Data insufficient |
| Walker et al. | UK prevalence of underlying conditions which increase the risk of severe COVID-19 disease: a point prevalence study using electronic health records. | Data insufficient |
| Morais-Almeida et al. | Update on asthma prevalence in severe COVID-19 patients. | Correspondence - no primary data |

**Supplementary Table 4: Risk of bias data**

| Authors | 1 | 2 | 3 | 4 | 5 | 6 | 7 | 8 | 9 | 10 | 11 | 12 | 13 | 14 | 15 | 16 | 17 | 18 | 19 | 20 | 21 | 22 | 23 | 24 | 25 | 26 | 27 | 28 | 29 | 30 | 31 | 32 | 33 | 34 | 35 | 36 | 37 | Overall risk of bias |
| --- | --- | --- | --- | --- | --- | --- | --- | --- | --- | --- | --- | --- | --- | --- | --- | --- | --- | --- | --- | --- | --- | --- | --- | --- | --- | --- | --- | --- | --- | --- | --- | --- | --- | --- | --- | --- | --- | --- |
| Atkins et al. | Y | Y | Y | N/A | Y | Y | N/A | N/A | N/A | N/A | N/A | N/A | Y | Y | UC | Y | Y | Y | N | Y | Y | Y | Y | Y | Y | Y | Y | Y | Y | Y | Y | N/A | Y | Y | Y | Y | Y | Low |
| Attaway et al. | Y | Y | Y | N/A | Y | Y | N/A | N/A | N/A | N/A | N/A | N/A | Y | Y | UC | Y | Y | Y | Y | Y | Y | Y | Y | Y | Y | Y | Y | Y | Y | Y | Y | N/A | Y | Y | Y | Y | Y | Low |
| Aveyard et al | Y | Y | Y | N/A | Y | Y | N/A | N/A | N/A | N/A | N/A | N/A | Y | Y | UC | Y | Y | Y | Y | Y | Y | Y | Y | Y | Y | Y | Y | Y | Y | Y | Y | N/A | Y | Y | Y | Y | Y | Low |
| Azoulay et al | Y | Y | Y | N/A | Y | Y | N/A | N/A | N/A | N/A | N/A | N/A | Y | Y | UC | Y | Y | Y | Y | Y | Y | Y | Y | Y | Y | Y | Y | Y | Y | Y | Y | N/A | Y | Y | Y | Y | Y | Low |
| Bloom et al. | Y | Y | Y | N/A | Y | Y | N/A | N/A | N/A | N/A | N/A | N/A | Y | Y | UC | Y | Y | Y | Y | Y | Y | Y | Y | Y | Y | Y | Y | Y | Y | Y | Y | N/A | Y | Y | Y | Y | Y | Low |
| Cellina et al. | Y | Y | Y | N/A | Y | Y | N/A | N/A | N/A | N/A | N/A | N/A | Y | Y | UC | Y | Y | Y | N | Y | Y | Y | Y | Y | Y | Y | Y | Y | Y | Y | Y | N/A | Y | Y | Y | Y | Y | Low |
| Choi et al. | Y | Y | Y | N/A | Y | Y | N/A | N/A | N/A | N/A | N/A | N/A | Y | Y | UC | Y | Y | Y | N | Y | Y | Y | Y | Y | Y | Y | Y | Y | Y | Y | Y | N/A | Y | Y | Y | Y | Y | Low |
| Choi et al. | Y | Y | Y | N/A | Y | Y | N/A | N/A | N/A | N/A | N/A | N/A | Y | Y | UC | Y | Y | Y | N | Y | Y | Y | Y | Y | Y | Y | Y | Y | Y | Y | Y | N/A | Y | Y | Y | Y | Y | Low |
| De Vito et al. | Y | Y | Y | N/A | Y | Y | N/A | N/A | N/A | N/A | N/A | N/A | Y | Y | UC | Y | Y | Y | N | Y | Y | Y | Y | Y | Y | Y | Y | Y | Y | Y | Y | N/A | Y | Y | Y | Y | Y | Low |
| De Vito et al. | Y | Y | Y | N/A | Y | Y | N/A | N/A | N/A | N/A | N/A | N/A | Y | Y | UC | Y | Y | Y | N | Y | Y | Y | Y | Y | Y | Y | Y | Y | Y | Y | Y | N/A | Y | Y | Y | Y | Y | Low |
| Giannouchos et al. | Y | Y | Y | N/A | Y | Y | N/A | N/A | N/A | N/A | N/A | N/A | Y | Y | UC | Y | Y | Y | N | Y | Y | Y | Y | Y | Y | Y | Y | Y | Y | Y | Y | N/A | Y | Y | Y | Y | Y | Low |
| Girardin et al. | Y | Y | Y | N/A | Y | Y | N/A | N/A | N/A | N/A | N/A | N/A | Y | Y | UC | Y | Y | Y | N | Y | Y | Y | Y | Y | Y | Y | Y | Y | Y | Y | Y | N/A | Y | Y | Y | Y | Y | Low |
| Grandbastien et al. | Y | Y | Y | N/A | Y | Y | N/A | N/A | N/A | N/A | N/A | N/A | Y | Y | UC | Y | Y | UC | UC | Y | Y | Y | Y | Y | Y | Y | Y | Y | Y | Y | Y | N/A | Y | Y | Y | Y | Y | Low |
| Grasselli et al. | Y | Y | Y | N/A | Y | Y | N/A | N/A | N/A | N/A | N/A | N/A | Y | Y | UC | Y | Y | Y | N | Y | Y | Y | Y | Y | Y | Y | Y | Y | Y | Y | Y | N/A | Y | Y | Y | Y | Y | Low |
| Guan et al | Y | Y | Y | N/A | Y | Y | N/A | N/A | N/A | N/A | N/A | N/A | Y | Y | UC | Y | Y | Y | N | Y | Y | Y | Y | Y | Y | Y | Y | Y | Y | Y | Y | N/A | Y | Y | Y | Y | Y | Low |
| Gupta et al. | Y | Y | Y | N/A | Y | Y | N/A | N/A | N/A | N/A | N/A | N/A | Y | Y | UC | Y | Y | UC | Y | Y | Y | Y | Y | Y | Y | Y | Y | Y | Y | Y | Y | N/A | Y | Y | Y | Y | Y | Low |
| Harrison et al. | Y | Y | Y | N/A | Y | Y | N/A | N/A | N/A | N/A | N/A | N/A | Y | Y | UC | Y | Y | UC | UC | Y | Y | Y | Y | Y | Y | Y | Y | Y | Y | Y | Y | N/A | Y | Y | Y | Y | Y | Low |
| Hernandez-Galdamez et al. | Y | Y | Y | N/A | Y | Y | N/A | N/A | N/A | N/A | N/A | N/A | Y | Y | UC | Y | Y | UC | N | Y | Y | Y | Y | Y | Y | Y | Y | Y | Y | Y | Y | N/A | Y | Y | Y | Y | Y | Low |
| Ho et al | Y | Y | Y | N/A | Y | Y | N/A | N/A | N/A | N/A | N/A | N/A | Y | Y | UC | Y | Y | Y | N | Y | Y | Y | Y | Y | Y | Y | Y | Y | Y | Y | Y | N/A | Y | Y | Y | Y | Y | Low |
| Hu et al | Y | Y | Y | N/A | Y | Y | N/A | N/A | N/A | N/A | N/A | N/A | Y | Y | UC | Y | Y | Y | N | Y | Y | Y | Y | Y | Y | Y | Y | Y | Y | Y | Y | N/A | Y | Y | Y | Y | Y | Low |
| Hu et al. | Y | Y | Y | N/A | Y | Y | N/A | N/A | N/A | N/A | N/A | N/A | Y | Y | UC | Y | Y | UC | N | Y | Y | Y | Y | Y | Y | Y | Y | Y | Y | Y | Y | N/A | Y | Y | Y | Y | Y | Low |
| Jiang et al. | Y | Y | Y | N/A | Y | Y | N/A | N/A | N/A | N/A | N/A | N/A | Y | Y | UC | Y | Y | Y | N | Y | Y | Y | Y | Y | Y | Y | Y | Y | Y | Y | Y | N/A | Y | Y | Y | Y | Y | Low |
| Kammar-Garcia et al. | Y | Y | Y | N/A | Y | Y | N/A | N/A | N/A | N/A | N/A | N/A | Y | Y | UC | Y | Y | Y | N | Y | Y | Y | Y | Y | Y | Y | Y | Y | N | UC | Y | N/A | Y | Y | Y | Y | Y | Medium |
| Lee et al. | Y | Y | Y | N/A | Y | Y | N/A | N/A | N/A | N/A | N/A | N/A | Y | Y | UC | Y | Y | Y | N | Y | Y | Y | Y | Y | Y | Y | Y | Y | Y | Y | Y | N/A | Y | Y | Y | Y | Y | Low |
| Li et al. | Y | Y | Y | N/A | Y | Y | N/A | N/A | N/A | N/A | N/A | N/A | Y | Y | UC | Y | Y | Y | N | Y | Y | Y | Y | Y | Y | Y | Y | Y | Y | Y | Y | N/A | Y | Y | Y | Y | Y | Low |
| Mahdavinia et al. | Y | Y | Y | N/A | Y | Y | N/A | N/A | N/A | N/A | N/A | N/A | Y | Y | UC | Y | Y | Y | N | Y | Y | Y | Y | Y | Y | Y | Y | Y | N | Y | Y | N/A | Y | Y | Y | Y | Y | Low |
| Martos-Benitez et al | Y | Y | Y | N/A | Y | Y | N/A | N/A | N/A | N/A | N/A | N/A | Y | Y | UC | Y | Y | Y | N | Y | Y | Y | Y | Y | Y | Y | Y | Y | Y | Y | Y | N/A | Y | Y | Y | Y | Y | Low |
| Murillo-Zamora et al | Y | Y | Y | N/A | Y | Y | N/A | N/A | N/A | N/A | N/A | N/A | Y | Y | UC | Y | Y | Y | N | Y | Y | Y | Y | Y | Y | Y | Y | Y | Y | Y | Y | N/A | Y | Y | Y | Y | Y | Low |
| Parra-Bracamonte et al. | Y | Y | Y | N/A | Y | Y | N/A | N/A | N/A | N/A | N/A | N/A | Y | Y | UC | Y | Y | Y | N | Y | Y | Y | Y | Y | Y | Y | Y | Y | N | Y | Y | N/A | Y | Y | Y | Y | Y | Low |
| Rosenthal et al | Y | Y | Y | N/A | Y | Y | N/A | N/A | N/A | N/A | N/A | N/A | Y | Y | UC | Y | Y | Y | N | Y | Y | Y | Y | Y | Y | Y | Y | Y | Y | Y | Y | N/A | Y | Y | Y | Y | Y | Low |
| Timerlake et al | Y | Y | Y | N/A | Y | Y | N/A | N/A | N/A | N/A | N/A | N/A | Y | Y | UC | Y | Y | Y | N | Y | Y | Y | Y | Y | Y | Y | Y | Y | Y | Y | Y | N/A | Y | Y | Y | Y | Y | Low |
| Wang et al. | Y | Y | Y | N/A | Y | Y | N/A | N/A | N/A | N/A | N/A | N/A | Y | Y | UC | Y | Y | Y | N | Y | Y | Y | Y | Y | Y | Y | Y | Y | Y | Y | Y | N/A | Y | Y | Y | Y | Y | Low |
| Wang et al. | Y | Y | Y | N/A | Y | Y | N/A | N/A | N/A | N/A | N/A | N/A | Y | Y | UC | Y | Y | Y | N | Y | Y | Y | Y | Y | Y | Y | Y | Y | Y | Y | Y | N/A | Y | Y | Y | Y | Y | Low |
| Wang et al. | Y | Y | Y | N/A | Y | Y | N/A | N/A | N/A | N/A | N/A | N/A | Y | Y | UC | Y | Y | Y | N | Y | Y | Y | Y | Y | Y | Y | Y | Y | Y | Y | Y | N/A | Y | Y | Y | Y | Y | Low |
| Wu et al. | Y | Y | Y | N/A | Y | Y | N/A | N/A | N/A | N/A | N/A | N/A | Y | Y | UC | Y | Y | Y | N | Y | Y | Y | Y | Y | Y | Y | Y | Y | N | Y | Y | N/A | Y | Y | Y | Y | Y | Low |
| Yoshida et al. | Y | Y | Y | N/A | Y | Y | N/A | N/A | N/A | N/A | N/A | N/A | Y | Y | UC | Y | Y | Y | N | Y | Y | Y | Y | Y | Y | Y | Y | Y | Y | Y | Y | N/A | Y | Y | Y | Y | Y | Low |
| Zhu et al. | Y | Y | Y | N/A | Y | Y | N/A | N/A | N/A | N/A | N/A | N/A | Y | Y | UC | Y | Y | UC | UC | Y | Y | Y | Y | Y | Y | Y | Y | Y | Y | Y | Y | N/A | Y | Y | Y | Y | Y | Low |

UC=unclear; 1=The source population or population of interest is adequately described for Key characteristics; 2= The sampling Frame and recruitment are adequately described, possibly including methods to identify the sample, place of recruitment and period of recruitment; 3= Inclusion and exclusion criteria are adequately described; 4= There is adequate participation in the study by eligible individuals; 5= The baseline sample is adequately described for key characteristics; 6= The study sample represents the population of interest on key characteristics, sufficient to limit potential bias of the observed relationship between the prognostic factor and outcome; 7= Response rate is adequate and is >80%; 8= Attempts to collect information on participants who dropped out of the study are described; 9= Reasons for loss to follow up are described; 10= Participants lost to follow up are adequately described for key characteristics; 11= There are no important differences between key characteristics and outcomes in participants who completed the study and those who did not; 12= Loss to follow up is not associated with key characteristics sufficient to limit potential bias to the observes relationship between the prognostic factor and the outcome; 13= A clear definition of description of the prognostic factors is provided; 14= Method of prognostic factor measurement is adequately valid and reliable to limit misclassification bias; 15= The prognostic factors measured are blinded for outcome measure; 16= Continuous variables are reported or appropriate cut offs are used; 17= The method and setting of measurement of PF is the same for all study participants; 18= More than 80% of the study sample has completed data for the PF variable; 19= Appropriate methods of imputation are used for missing PF data; 20= PF is adequately measured in study participants to sufficiently limit potential bias; 21= A clear definition of outcome is provided; 22= The method of outcome measurement used is valid and reliable to limit misclassification bias; 23= The method and setting of outcome measurement is the same for all study participants; 24= Outcome of Interest is adequately measured in study participants to sufficiently limit potential bias; 25= All important confounders are measured; 26= Clear definitions of the important confounders measured are provided; 27=The method and setting of confounding measurement are the same for all study participants; 28= Important potential confounders are accounted for in the study design; 29= Important potential confounders are accounted for in the analysis; 30= Important potential confounders are appropriately accounted for, limiting potential bias with respect to the relationship between PF and outcome; 31= There is sufficient presentation of data to assess the adequacy of the analysis; 32= The strategy for model building is appropriate and is based on a conceptual framework or model; 33= The selected statistical model is adequate for the design of the study; 34= There is description of the association of the prognostic factor and the outcome, including information about the statistical significance; 35= Continuous variables are reported or cut-off points are used; 36= The is no selective reporting of results; 37= The statistical analysis is appropriate for the design of the study, limiting potential for presentation of invalid or spurious results
